# Development and validation of matrix metalloproteinase for predicting prognosis and tumour microenvironment immune profiles in uterine corpus endometrial carcinoma

**DOI:** 10.7150/jca.91277

**Published:** 2024-05-30

**Authors:** Huancheng Su, Yutong Yang, Chu Li, Jinpeng Li, Huihui Lv, Xiaoyao Jia, Jiaolin Yang, Jing Lei, Xia Li, Hongrui Guo, Zhe Wang, Sanyuan Zhang

**Affiliations:** 1 Shanxi Medical University, Taiyuan 030001, China.; 2 Department of Gynecology, First Hospital of Shanxi Medical University, Taiyuan 030001, China.; 3 College of nursing, Shanxi medical university, Taiyuan 030001, China.; 4 Department of Tuina, Shenzhen Traditional Chinese Medicine Hospital, The Fourth Clinical Medical College of Guangzhou University of Chinese Medicine, China.

**Keywords:** Matrix metalloproteinases (MMPs), Endometrial cancer (EC), Tumour microenvironment (TME), Tumour mutation burden (TMB), Immune infiltration, Immune checkpoint

## Abstract

**Background**: Matrix metalloproteinases (MMPs) are involved in many processes of tumour progression and invasion. However, few studies have analysed the effects of MMP expression patterns on endometrial cancer (EC) development from the perspective of the tumour microenvironment (TME). we quantified MMP expression in individual by constructing an MMP score and found MMP score effectively predict the prognosis of EC patients.

**Methods**: MMPs expression profiles were determined based on the differential expression of 12 MMP-related regulators. Principal component analysis (PCA) was used to construct an MMP scoring system which can quantify the MMPs expression patterns individually of EC patients. Kaplan-Meier analysis, the log-rank test, and time-dependent receiver operating characteristic (ROC) curve analysis were used to evaluate the value of MMPs expression in predicting prognosis. Single-cell RNA sequencing (scRNA-seq) dataset was used to verify correlation between MMPs and progression of EC. Gene Ontology (GO) analysis was used to investigate the pathways and functions underlying MMPs expression. Tumour immune dysfunction, exclusion prediction, and pharmacotherapy response analyses were performed to assess the potential response to pharmacotherapy based on MMPs patterns.

**Results**: We downloaded the MMPs expression data, somatic mutation data and corresponding clinical information of EC patients from the TCGA website and ICGC portal. Based on the MMP-related differentially expressed genes (DEGs), the MMP score was constructed, and EC patients were divided into high and low MMP score groups. There was a positive correlation between MMP score and prognosis of EC patients. Patients with high MMP scores had better prognosis, more abundant immune cell infiltration and stronger antitumoor immunity. Although prognosis is worse with the lower group than the high, patients with low MMP score had better response to immunotherapy, which means they could prolong the survival time through Immunological checkpoint blockade (ICB) therapy. scRNA-seq analysis identified significant heterogeneity between MMP score and classical pathways in EC.

**Conclusion**: Our work indicates that the MMP score could be a potential tool to evaluate MMP expression patterns, immune cell infiltration, response to pharmacotherapy, clinicopathological features, and survival outcomes in EC. This will provide the more effective guide to select immunotherapeutic strategies of EC in the future.

## 1. Introduction

Endometrial cancer (EC) has the second highest incidence among types of gynaecologic cancer 1. Ninety percent of women complain that they suffer the symptom of postmenopausal bleeding (PMB), however, only around ten percent of them have a chance of developing into EC 2, which suggests that PMB is not a specific indicator of EC diagnosis. Similarly, laboratory tests for evaluation of EC, such as cytology and transvaginal ultrasonography, lack specificity. Thus, there is a necessity for accurate screening tools to identify early EC patients. Recently, numerous studies have demonstrated a correlation between matrix metalloproteinases (MMPs) and EC 3, 4. Therefore, the functional roles and regulatory mechanisms of MMPs in EC need to be further investigated, and it is crucial to elucidate the association between MMPs and prognosis of EC.

The tumour microenvironment (TME) is the cellular environment in which tumours or tumour stem cells exist, including tumour cells, adipocytes, fibroblasts, lymphocytes, dendritic cells, cancer-associated fibroblasts (CAF), and tumour vasculature, and it is widely associated with tumourigenesis 5, 6. These cells interact with the circulatory and lymphatic systems to promote tumourigenesis and cancer progression. CAF of different origins contribute to the heterogeneity of tumour cells and exert functional effects on tumours via various mechanisms 7-9. Oncogenic signalling mediates tumour immune escape, including decreased effective immunocyte infiltration and function and increased levels of immunosuppressive cells in the TME 10-13. The two important stages of tumour development are degradation of the basement membrane and invasion of tumour cells into the surrounding tissue. Metastasis of cancer cells is a complex multistep process, involving changes in intercellular adhesion, degradation of the extracellular matrix (ECM) and basement membrane, detachment of tumour cells in situ, and extensive infiltration of proteolytic enzymes into lymphatic or blood vessels 5, 6. MMPs are diverse enzymes involved in ECM, which are primarily responsible for the reshaping of tissue by degrading collagen IV and laminin 8, 9. Various MMPs are produced by tumours and tumour-related cells. In the TME, *MMP-1, MMP-2* and *MMP-3,* secreted by fibroblasts, are essential mediators of tumour angiogenesis and progression. *MMP-9* secreted by neutrophils, mast cells, and macrophages degrades the main components of the basement membrane to promote tumour invasion 14, 15. Fibroblasts and tumour cells secrete *MMP-13*, *MMP-7*, and *MMP-14*. *MMP-13* promotes tumour angiogenesis 16, *MMP-7* degrades HB-EGF and E-cadherin in the basement membrane 17, 18, and *MMP-14* degrades CD-44 and electron-cadherin in the basement membrane 19, which together play a vital role in tumour invasion. *MMP-10* is highly expressed in squamous cells and promotes the recruitment of infiltrating cells by remodelling ECM. *MMP-10* can also upregulate the expression of *MMP-7*, *MMP-9,* and *MMP-13*, which are critical for tumour progression 20. Recently, Zhang *et al.* reported significantly higher *MMP-7* expression in ovarian cancer tissue than in normal ovarian tissue 21. To summarise, MMPs play an important role in regulating tumour metastasis.

However, it is still unclear whether the expression levels of MMPs are related to the occurrence and progression of EC, and whether they have a predictive effect on the prognosis of EC. In this study, we used The Cancer Genome Atlas (TCGA) and International Cancer Genome Consortium (ICGC) databases to analyse differentially expressed genes (DEGs) of MMPs. Unsupervised clustering was used to identify two expression patterns (MMP cluster A and MMP cluster B). We performed a series of correlation analysis of the infiltration of immune cells under the two patterns and found that MMP clusters A and B were highly consistent with immune rejection and immune inflammation phenotypes, respectively. Furthermore, in consideration of individual heterogeneity, we constructed the MMP score to predict prognosis, which is of great significance for guiding the clinical treatment of EC patients.

## 2. Methods

### 2.1 EC dataset source and preprocessing

We downloaded the MMPs expression data, somatic mutation data and corresponding clinical information of EC patients from the TCGA website (https://portal.gdc.cancer.gov/projects/TCGA-UCEC) and ICGC portal (https://dcc.icgc.org/projects/UCEC-US) up to July 31, 2023. In total, eligible TCGA-UCEC (The Cancer Genome Atlas-Uterine Corpus Endometrial Carcinoma) and UCEC-US (Uterine Corpus Endometrial Carcinoma- TCGA, US) were gathered in this study for further analysis. The gene expression profiles were normalized using the scale method provided in the 'limma' package (version 3.58.1), and normalized read count values were used.

### 2.2 Unsupervised clustering and consensus clustering analysis

A total of 12 MMP-related genes were extracted from TCGA datasets to identify the different expression patterns mediated by 12 MMPs. The 12 MMPs were *MMP2*, *MMP-3*, *MMP-10*, *MMP-9*, *MMP-11*, *MMP-12*, *MMP-13*, *MMP-15*, *MMP-19*, *MMP-23B*, *MMP-24*, and *MMP-28*. Unsupervised clustering analysis was used to identify distinct MMP-related gene patterns based on their expression, and patients were classified for further analysis. The number of clusters and their stabilities were determined using a consensus clustering algorithm. The 'ConsensusClusterPlus' package (version 1.66.0) was used to perform the above steps and 1000 repetitions were conducted to guarantee the stability of the classification.

Geneset variation analysis (GSVA) enrichment analysis, conducted using the 'GSVA' package (version 1.50.0), was performed to investigate the differences in biological processes between MMP patterns. GSVA, a non-parametric and unsupervised method, is commonly employed to estimate the variation in pathways and biological process activities in the samples of an expression dataset. Kyoto Encyclopedia of Genes and Genomes (KEGG) analysis is a useful tool to search mediated mechanisms and pathway 22. The gene sets of 'c2.cp.kegg.v6.2.Symbols' were downloaded from the MSigDB database for analysis. Adjusted P-values of <0.05 were considered to indicate statistical significance. The 'clusterProfiler' package (version 4.1.0) was used to perform functional annotation of MMP-related genes with a cut-off value of false discovery rate <0.05.

### 2.3 Identification of DEGs between MMPs gene distinct phenotypes and TME cell infiltration estimation

To identify MMP-related genes, we classified the patients into two distinct MMP patterns based on MMP gene expression. The empirical Bayesian approach of the 'limma' package was used to identify DEGs between different patterns. The significance criteria for determining DEGs were set at an adjusted *P* value of < 0.001. We used a single-sample gene set enrichment analysis (ssGSEA) algorithm to quantify the relative abundance of each type of cell infiltration in the EC TME 23. The gene set for marking each TME infiltrating immune cell type was obtained from the study by Charoentong, which stored various human immune cell subtypes, including activated CD8 T cells, activated dendritic cells, macrophages, natural killer T cells, and regulatory T cells. The enrichment scores caculated by ssGSEA, were used to represent the relative abundance of each TME-infiltrating cell in each sample.

### 2.4 Design and generation of an MMP score

The MMP gene signature, a set of scoring systems termed the MMP score, was constructed to evaluate the MMP patterns in individual patients with EC. The MMP gene signature was established as follows:

First, we identified DEGs from two MMP clusters which were normalized, and extracted the overlapping genes. By adopting an unsupervised clustering method to analyse overlapping DEGs, patients were classified into three groups for further analysis. A consensus-clustering algorithm was used to define the number of gene clusters and their stability.

Second, we performed prognostic analysis for each gene in the signature using a univariate Cox regression model. Genes with significant prognostic values were extracted for further analysis. We conducted principal component analysis (PCA) to construct an MMP-relevant gene signature. Principal components 1 and 2 were selected as signature scores 24.

MMP score = Σ(PC1*_i_*+PC2*_i_*)

Where i is the expression of MMP phenotype-related genes.

### 2.5 Assessing the response to immunotherapy and drug sensitivity

Tumor Immune Dysfunction and Exclusion (TIDE) is a computational method that models tumour immune evasion. We used TIDE to predict the response to Immunological checkpoint blockade (ICB) in different MMP score groups (http://tide.dfci.harvard.edu/). The 'limma' package was used to calculate TIDE scores in different MMP score groups, and the 'pRRophetic' package was used to analyse the expression profile of the drug sensitivity analysis.

### 2.6 Correlation between the MMP gene score and other related biological processes or clinic features

We performed a correlation analysis to examine the association between the MMP gene signature and some related biological pathways or clinical features, including (1) immune-checkpoints such as CD 44, CD 274 and PDCD1; (2) microsatellites, such as microsatellite-stable (MSS), microsatellite instability-low (MSI-L), microsatellite instability-high (MSI-H); (3) tumour mutational burden (TMB); (4) age; (5) tumour grade; (6) several drugs including thapisgargin, doxorubicin, rapamycin, nilotinib and temsirolimus; (7) ESTIMATES score; (8) immune score; (9) stromal score; (10) tumour purity.

### 2.7 Immunohistochemistry of six samples and Quantitative real-time polymerase chain reaction (qRT-PCR)

Endometrial tissue samples were collected from three EC patients and three normal women respectively in the First Hospital of Shanxi medical University. We selected samples based on the findings of histopathology, which was performed by a pathologist with More than two years of clinical experience. To improve accuracy of diagnosis for endometrial tissue, forceps were used to remove two additional pieces of tissue (approximately 5 mm each). EC patients were included with histologically proven endometrial carcinoma, but normal women were included with normal endometrial tissue using the same methods. Additionally, following samples were excluded: (1) history of hysterectomy; (2) pregnancy; (3) combined with other site malignancies; (4) severe cardiovascular and cerebrovascular diseases; (5) no prior chemoradiotherapy or immunotherapy. This study was approved by the ethics committee (number: 2021-K-K221) and all enrolled patients signed informed consent.

We performed qRT-PCR after collection of samples. Firstly, the tissue was washed with physiological saline and placed in a solution containing RNA preservation and tissue fixation. Secondly, samples were stored overnight in a 4°C refrigerator and then transferred to a -20°C refrigerator for storage at room temperature for hematoxylin and eosin samples. Thirdly, total RNA was extracted from normal and EC tissue using TRlzol reagent (Invitrogen) according to the manufacturer's instructions. The primers were synthesized by Sangon Biotech (Shanghai, China). The housekeeping gene GADPH was used as an internal control. The primers used are listed as follows: *MMP-3* forward, 5'-GGTGAGGACACCAGCATGAA-3' and reverse, 5'-TCAGGGGGAGTCCATAGAG-3'; *MMP-10* forward, 5'-ACAAGGATCTTCCCAGCAAT-3' and reverse, 5'-AGGAGCTGAAGTGACCAACG-3'; *MMP-11* forward, 5'-GATCGACTTCGCCAGGTACT-3' and reverse, 5'-TTTCACCGTCGTACACCCAG-3'; *MMP-12* forward, 5'-TTTCACCGTCGTACACCCAG-3' and reverse, 5'-TTTCCCACGGTAGTGACAGC-3'; *MMP-13* forward, 5'-ATGCAGCAAGCTCCATGACT-3' and reverse, 5'-ATGCAGCAAGCTCCATGACT-3'; *MMP-19* forward, 5'-CCAGTAGCGGTCACCTTTGA-3' and reverse, 5'-AGTACCCGGAGCCCCTTAAA-3'. All reactions were conducted on Roche LightCycler 96PCR Machine (Roche, Mannheim, Germany) using the following cycling parameters: step 1: denaturation at 94°C for 30 seconds; step 2: 40 cycles of 94°C for 5 seconds and 60°C for 30 seconds. Gene expression was calculated using the ΔΔCt method. All data represent the average of three replicates.

### 2.8 Single-cell RNA sequencing (scRNA-seq) dataset processing and analysis

ScRNA-seq dataset was performed on samples of a normal and an EC patients' endometrial tissue. The inclusion and exclusion criteria are the same as in section 2.7. scRNA-seq data preprocessing raw reads in the .fastq files of human endometrial cells were processed in the Cell Ranger Software Suite (10x Genomics Cell Ranger 4.0.0) using refdata-gex-GRCh38-2020-A as reference to map reads on the human genome (GRCh38/hg38), and generate the unique molecular identifier (UMI) matrices 25. The Cell Ranger outputs were imported into Seurat by the 'Read10X' function 26. Among each sample, cells with UMI counts above upper 10% are removed. Then cells with fewer than 500 UMI counts detected or >40% mitochondrial UMI counts were filtered out. Finally, genes expressed in less than 10 cells were also removed. The 'Seurat' package (version 4.0) was applied in the quality control procedure. To eliminate differences in gene expression between cells based on count data, a global scaling normalization method, LogNormalize, was applied to normalize the measurements of characteristic expression per cell as well as the total expression. Data were normalized using the ScaleData function in the 'Seurat' package (version 4.0). The data were scaled according to a linear transformation to ensure that each gene was given the same weight, with a mean of 0 and a variance of 1. To reduce the computational burden and noise in the data, PCA was used for initial dimensionality reduction. The annotated information for each cell in the dataset reported was visualized using uniform manifold approximation and projection. K-nearest-neighbour graphs were constructed using the FindNeighbors function based on the Euclidean distance in the PCA space, whereas cells were clustered using the Louvain algorithm. The annotated information for each cell in the dataset reported was visualized using uniform manifold approximation and projection (UMAP).

### 2.9 Statistical analysis

Data were analysed using R (version 3.6.1) and R Bioconductor packages. One-way analysis of variance (ANOVA) and the Kruskal-Wallis test were used to compare the differences among three or more groups. Spearman's and distance correlation analyses were used to calculate the correlation coefficients between the expression of MMP regulators and the infiltrating immune cells. The 'survminer' package (version 0.4.7) was used to determine the optimal cut-off point of the MMP score for predicting prognosis, and patients were divided into high and low MMP score groups. Survival curves were generated using the Kaplan-Meier method, and log-rank tests were used to identify the significance of differences. A univariate Cox regression model was used to calculate the Hazard Ratios (HRs) for MMP regulators and MMP phenotype-related genes. Multivariate Cox regression analysis was used to evaluate the independent prognostic factors. The specificity and sensitivity of the MMP score were assessed using a receiver operating characteristic curve (ROC), and the area under the curve was quantified using the 'pROC' package (version 1.78.0). Waterfall plots representing the mutation landscapes of the high and low MMP score groups were created using the waterfall function of 'maftools' package (version 2.18.0). All tests were bilateral, and *P* < 0.05 was considered statistically significant. Power calculations were performed using 'rstatix' package (version 0.7.2) at a significance level of 0.05.

## 3. Results

### 3.1 MMPs genetic variation and the expression landscape in EC

**Figure 1:**
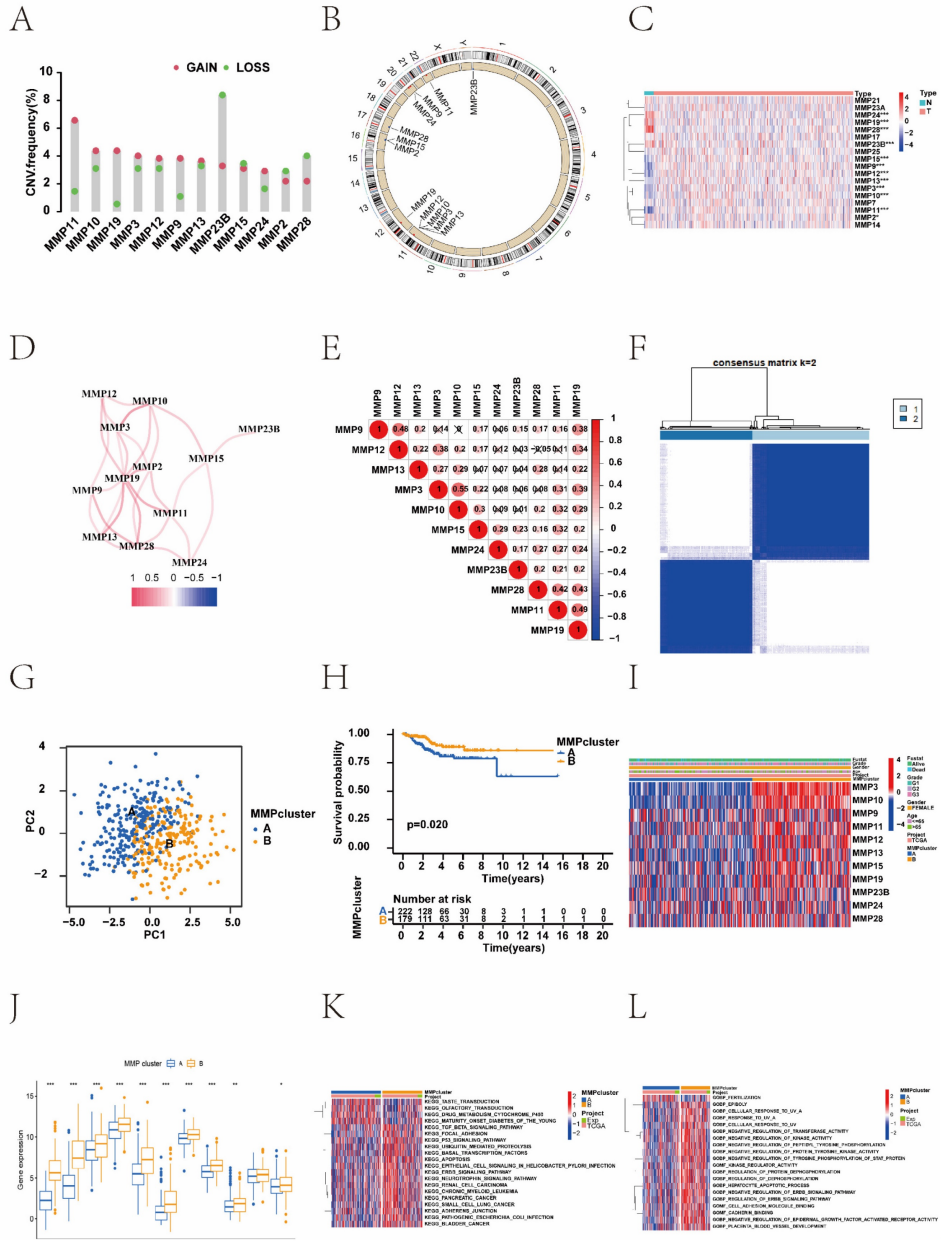
MMPs genetic variation and expression landscape in EC. (A) The CNV variation frequency of MMPs from TCGA-UCEC and UCEC-US cohort. The height of the column represents the alteration frequency. The deletion frequency, green dot; The amplification frequency, red dot. (B) The location of CNV alteration of MMPs on 23 chromosomes using TCGA-UCEC. The deletion frequency, blue dot; The amplification frequency, red dot. (C) Expression heat map of the 11 MMPs in normal and EC samples in TCGA-UCEC cohort. Tumour, red; Normal, blue. (D) The interaction of expression on 11 MMPs in EC. (E) The Pearson correlation among 11 MMPs. The positive correlation is marked with red, and negative correlation is marked with blue. The size of circle represents the absolute value of correlation coefficients. (F) Consensus matrices of the significant MMPs for k = 2. (G) Principal component analysis for the transcriptome profiles of MMP expression patterns, showing a remarkable difference in the transcriptome between different expression patterns. (H) Survival difference among two MMP expression patterns (*P* = 0.020, Kruskal-Wallis test). MMP cluster-A, yellow; MMP cluster-B, blue. (I) Unsupervised clustering of 11 MMP regulators in the TCGA-UCEC cohort identified a significant difference in the expression of regulators among the two expression patterns. The MMP clusters, TCGA project, age, sex, clinical stage, and survival status were used as patient annotations. Red, high expression of regulators; blue, low expression of regulators. (J) Differences of 11 MMPs gene expression between MMP cluster A and MMP cluster B. MMP cluster-A, blue; MMP cluster-B, yellow, the upper and lower ends of the boxes represent the interquartile range of values. The lines in the boxes represent the median value, and black dots showed outliers. The asterisks represented the statistical *P* value (*P < 0.05; **P < 0.01; ***P < 0.001). (K) The biological behaviour between these different MMPs expression patterns were analysed by KEEG enrichment using TCGA-UCEC and UCEC-US cohort; red represents activated pathways and blue represents inhibited pathways. (L) GO analysis showing the Cellular Component, Molecular Function and distinct biological processes in in distinct MMP cluster expression patterns using TCGA-UCEC and UCEC-US cohort. The heatmap was used to visualize these biological processes, and red represents activated pathways and blue represents inhibited pathways. MMP, matrix metalloproteinases; EC, endometrial cancer; CNV, copy number variation; TCGA-UCEC, The Cancer Genome Atlas-Uterine Corpus Endometrial Carcinoma; UCEC-US, Uterine Corpus Endometrial Carcinoma-TCGA, US; KEGG, Kyoto Encyclopedia of Genes and Genomes; GO, gene ontology.

In this study, to investigate the relationship between MMPs CNV and expression, we systematically profiled the prevalence of the MMPs CNV and somatic mutations in EC. Investigation of CNV alteration frequency showed prevalent CNV alterations in 12 regulators (Figure 1A). *MMP-23B*, *MMP-24*, *MMP-2* and *MMP-28* showed CNV deletions, but other genes expressed widespread CNV amplification. The chromosomal locations of the MMPs are shown in Figure 1B. The results showed that a change in CNV may be the main factor leading to the disruption of MMPs expression. There were significant differences in the expression levels of the MMPs between patients with EC and normal controls (Figure 1C and Supplementary Figure 1). The correlation network diagram depicted the relationship between MMP interactions and further confirmed the ubiquitous correlation between the 12 MMPs (Figure 1D). We examined the Pearson correlation between the 11 MMPs associated with EC using Spearman's correlation analysis and found a positive correlation among MMPs (Figure 1E). To explore the relationship between biological behaviours and the genes contained in the CNV in EC, the KEGG and GO functional enrichment were performed. The results showed that those genes were enriched in metalloendopeptidase activity, metallopeptidase activity, endopeptidase activity, and IL-17 signalling pathway (Supplementary Figure 2A and 2B). In the TCGA cohort, we categorised a series of patients with different MMP expression patterns according to the expression of 12 MMPs. Two distinct expression patterns were identified using unsupervised clustering (Figure 1F). These patterns were named MMP clusters A and B according to the results of PCA (Figure 1G). Prognostic analysis of the two MMP expression subtypes revealed that MMP cluster B expressed more prominent survival advantage than cluster A (*P* = 0.020) (Figure 1H). To further explore the characteristics of MMP expression patterns, we conducted unsupervised clustering of 11 MMP regulators in TCGA cohort, including demographic and clinical data such as age, clinical stage, and survival status. The heat map not only revealed the characteristics of different clinical traits in the two MMP expression patterns but also their correlation with the expression of MMP regulators. There was a significant difference in the MMP-related gene transcriptional profiles between the two MMP expression patterns; most MMP regulators were downregulated in cluster A but MMPs were highly expressed in cluster B (Figure 1I, J).

The biological behaviours of MMPs were analysed using KEEG enrichment based on TCGA-UCEC and UCEC-US cohorts. The KEGG pathways of MMP cluster A were significantly enriched in 'drug metabolism cytochrome P450', and 'maturity onset diabetes of the young'. Many KEGG pathways of MMP cluster B were significantly enriched, such as the 'neurotrophin signalling pathway', 'renal cell carcinoma', 'chronic myeloid leukaemia', and 'pancreatic cancer'. To further investigate the potential biological behaviour of MMP gene patterns, we performed GO enrichment analysis for these gene sets. Notably, the KEGG pathways of the MMP cluster B in GO terms were profuse such as: 'negative regulation of tyrosine phosphorylation of stat protein', 'kinase regulator activity', 'regulation of protein dephosphorylation', and 'regulation of dephosphorylation' (Figure 1K, L).

### 3.2 Generation of MMP gene signatures and exploration of its clinical immune relevance

**Figure 2:**
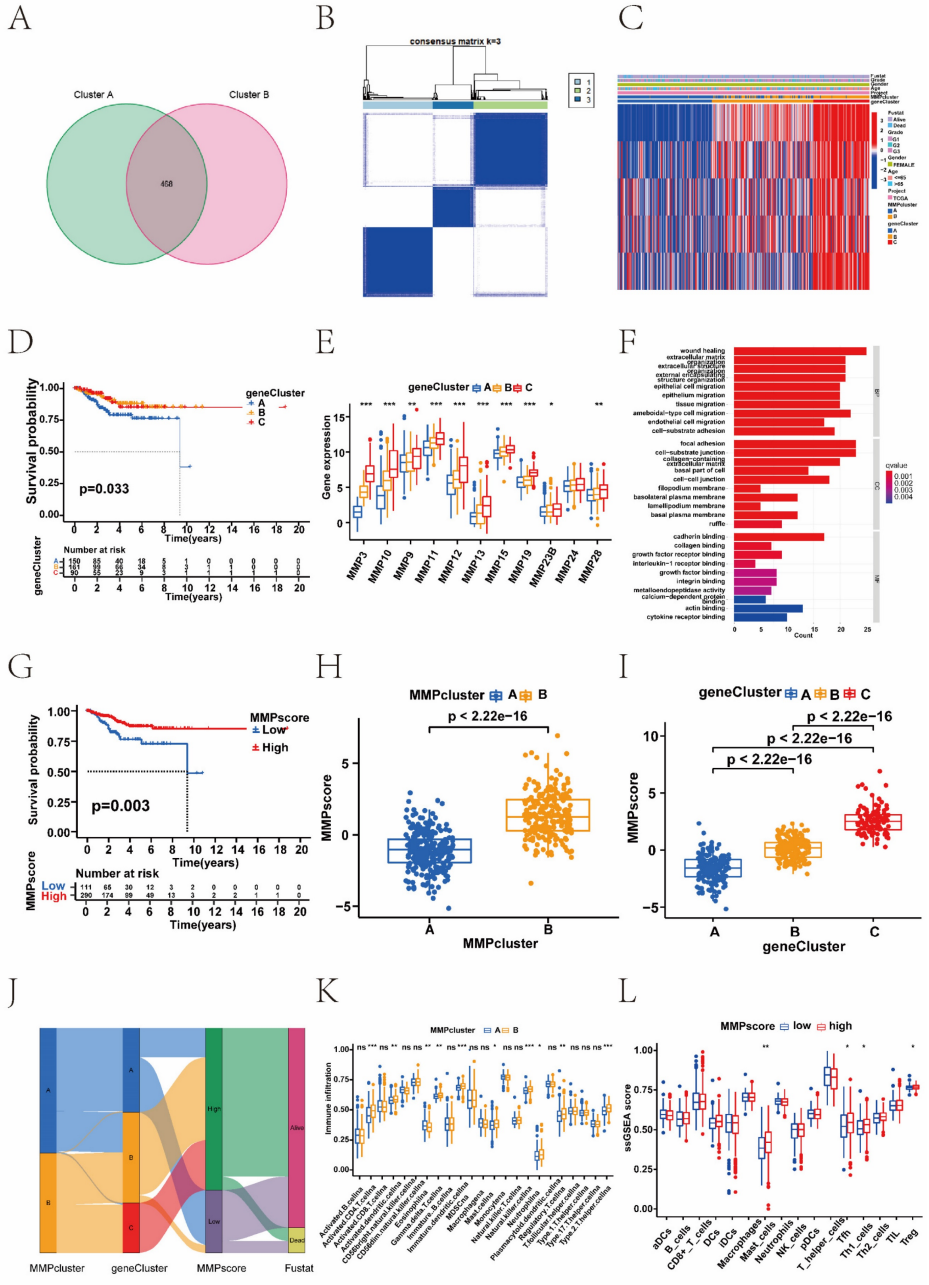
Construction of MMP gene signatures and functional annotation. (A) Four hundred and sixty-eight MMP-related DEGs between two MMP clusters are shown in the Venn diagram. (B) Three different genomic subtypes identified by unsupervised clustering based on the overlapping MMP phenotype-related DEGs (MMP gene cluster A, MMP gene cluster B, and MMP gene cluster C). (C) Unsupervised clustering of overlapping MMP phenotype-related DEGs identified three significantly different genomic subtypes. (D) survival difference among three MMP gene clusters (*P* = 0.033, Kruskal-Wallis test). Blue, MMP gene cluster A; yellow, MMP gene cluster B, and red, MMP gene cluster C. (E) Differences in the gene expression of 11 MMPs between MMP cluster A, MMP cluster B, and MMP cluster C. The thick line represents the median value. The bottom and top of the boxes were the 25th and 75th percentiles (interquartile range). The statistical difference of three gene clusters was compared through the Kruskal-Wallis H test. (F) The GO enrichment analysis is based on the overlapping MMP phenotype-related genes. The colour depth of the bar chart indicates the number of genes enriched. The length of the bar chart indicates the count of genes enriched. BP, biological process; CC, cellular component; MF, molecular function. (G) Survival analysis based on the MMP score patient groups in the TCGA cohort (Log-rank test, *P* < 0.003). (H) Differences in the MMP score among the two MMP clusters in the TCGA UCEC cohort (*P <* 0.001, Kruskal-Wallis test). (I) Differences in the MMP score among the three MMP gene clusters in the TCGA UCEC cohort (*P <* 0.001, Kruskal-Wallis test). (J) Sankey diagram demonstrating the relationship between MMP cluster, MMP gene cluster, MMP score, and survival status. (K) Correlation between immune cells and the MMP score. Infiltrating immune cell analysis based on the MMP cluster. The abundance of infiltrating immune cells was different among the two MMP clusters. (L) Differences in immune infiltrating cells between high MMP score and low MMP score groups in the ssGSEA. Blue, low MMP score group; red, high MMP score group. DEGs, differentially expressed genes; TCGA, The Cancer Genome Atlas; ssGSEA, single-sample gene set enrichment analysis.

To investigate the potential biological behaviour of each MMP expression pattern, we identified 468 DEGs associated with the MMP phenotypes (Figure 2A). To further validate the regulatory mechanisms of MMPs, we performed a consistent clustering analysis of the screened genes, which revealed three distinct MMPs genomic phenotypes in contrast to the clustered grouping of MMP expression patterns. We named three clusters as MMP gene clusters A, B, and C (Figure 2B). To explore the correlation between MMP expression genomic phenotypes, the clinical traits and biological behaviours of EC patients, unsupervised clustering analysis of MMP phenotype-related DEGs and clinical traits, such as age, clinical stage, and survival status, was conducted. Three distinct MMP gene clusters were identified, and 403 patients with EC were divided into three different genomic subtypes.

As shown in the heatmap, the three distinct gene clusters were characterized by different signature genes, and there were more downregulated genes in Cluster A than in the other gene clusters (Figure 2C and 2E). These results demonstrated that two MMP expression patterns were present in ECs and were closely related to the clinicopathological characteristics. Survival analysis revealed significant differences among the three gene clusters (*P* = 0. 033); EC patients in gene cluster C were associated with better prognosis. In contrast, patients in Cluster A had a poorer prognosis (Figure 2D). The difference in MMP gene expression between clusters A, B, and C is shown, where the majority of MMP genes were highly expressed in cluster C (Figure 2E). This suggested the existence of two distinct MMP expression patterns in EC. We performed GO analysis of different MMP gene clusters and found that they were significantly enriched in the ECM, collagen, and extracellular structural tissue (Figure 2F). Above analyses were based on the entire cohort. To further explore the heterogeneity and complexity of MMP expression, we constructed a set of scoring models based on these phenotype-related genes and called the MMP score, which conduced to quantify the MMP expression in individual tumour cells and to predict treatment response and prognosis of EC patients. The Kruskal-Wallis test revealed associations not only between the MMP clusters and MMP score, but also between the MMP gene clusters and MMP score. Next, we sought to determine the value of the MMP score in predicting patient prognosis. Patients were divided into the low or high MMP score group using a cut-off value of *P* < 0.05. Kaplan-Meier curves showed that patients with high MMP scores had a significant survival benefit (*P <* 0.05) (Figure 2G). Compared to the other clusters, MMP cluster A showed a significantly lower MMP score, whereas MMP cluster B showed a high median score (*P <* 0.05) (Figure 2H). Among three gene clusters, MMP gene cluster C had the highest median score, whereas MMP gene cluster A had the lowest (Figure 2I). MMP cluster B focused mainly on MMP gene clusters B and C, had higher MMP score and proportion of patients at an advanced clinical stage (Figure 2J).

In addition, we observed significantly different levels of immune cell infiltration in the two MMP clusters. To investigate the role of MMP expression in immune cell infiltration in the TME, we first compared immune cell characteristics among different MMP clusters. The two types of MMP clusters were significantly correlated with infiltration of the activated dendritic cells, activated CD4^+^ T cells, eosinophilna, gamma delta T cells, immature dendritic cells, macrophagena, natural killer cells, regulatory T cells, and type II T helper cells. MMP cluster B is remarkably abundant in innate immune cells, including immature dendritic cells, activated CD4^+^ T cells, natural killer cells, and type II T helper cells (Figure 2K). Considering the role of immune cell infiltration in tumour occurrence and development and its prognostic impact, we analysed the correlation between survival and the ssGSEA scores of 22 types of immune cells. As indicated by the heatmap, there was no significant correlation between survival status and the ssGSEA score of immune cells other than plasma cells (Supplementary Figure 2C). Further analysis by ssGSEA revealed that different MMP scores were significantly associated with high and low levels of immunological function and immune infiltration in the tumour tissue (Figure 2L, and Supplementary Figure 2D). Therefore, there was a remarkable difference of immune cell expression with MMP clusters and MMP score. The high MMP score group generally had higher immune cell scores, including those of mast cells, T helper cells, type I helper cells, and regulatory T cells.

### 3.3 The MMP score activates immune infiltration

**Figure 3:**
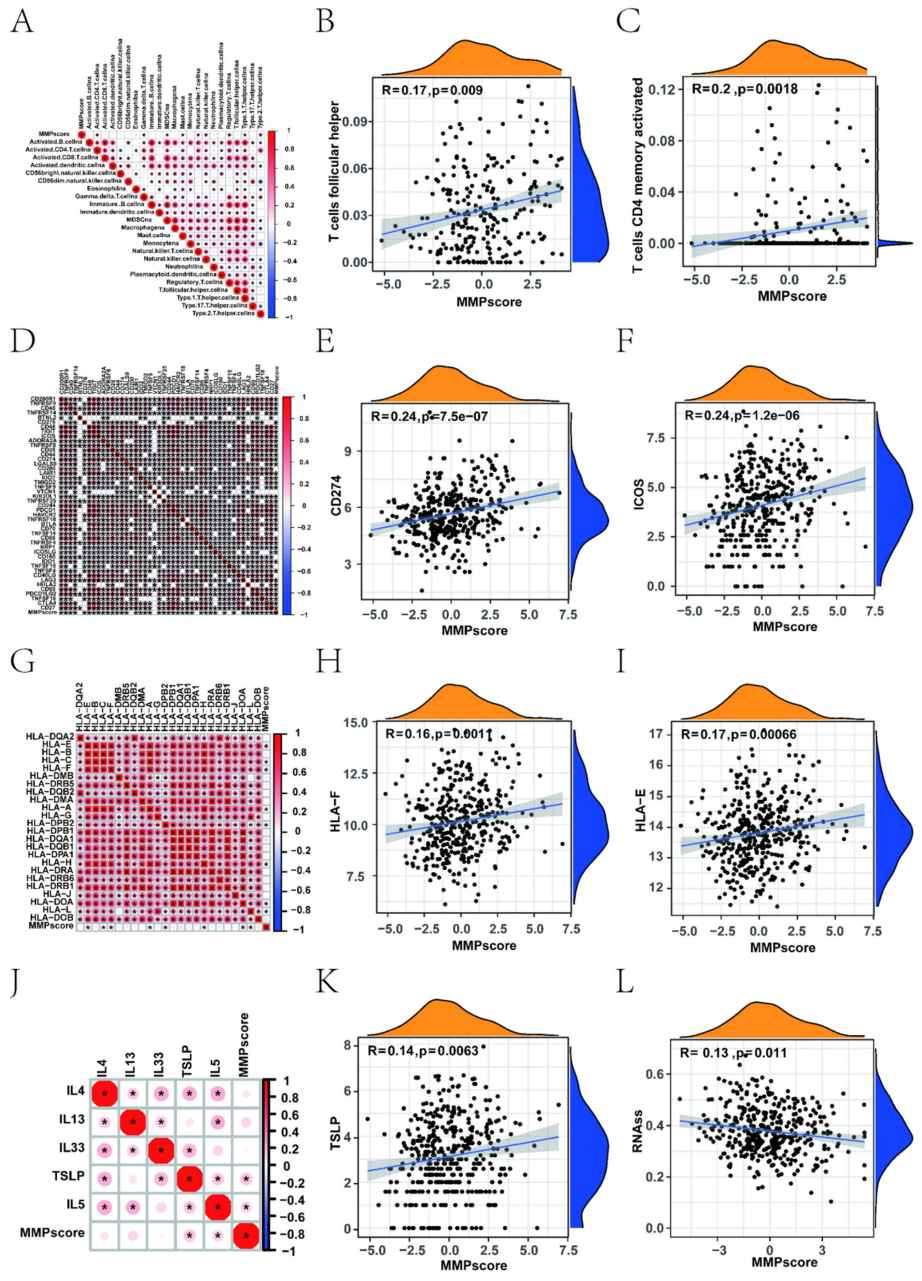
Correlation between different immune cells and the MMP score of EC patients. (A) Spearman's correlation analysis was used to analyse the correlation between the MMP score and known gene signatures in the TCGA UCEC cohort. The size of the dots represents the strength of the correlation. Red, positive correlation; blue, negative correlation. (B) Correlation between MMP score and T cells follicular helper in EC. R = 0.17, *P* = 0.009. (C) Correlation between MMP score and T cells CD4 memory activated in EC. R = 0.2, *P* = 0.0018. (D) Correlation between the MMP score and immune checkpoints. (E) Correlation between the MMP score and CD274 in EC. R = 0.24, *p* < 0.001. (F) Correlation between the MMP score and ICOS in EC. R = 0. 24, *P* < 0.001. (G) Correlation between the MMP score and HLA. (H) Correlation between the MMP score and HLA-E in EC. R = 0.17, *P* = 00066. (I) Correlation between the MMP score and HLA-F in EC. R = 0.16, *P* = 0. 0011. (J) Correlation between the MMP score and interleukins in EC. (K) Correlation between the MMP score and TSLP in EC. R = 0.14, *P* = 0063. (L) Correlation between RNAss and the MMP score in EC. R = 0.13, *P* = 0.011. HLA, human leukocyte antigens.

To better characterise the correlation between immune cells and the MMP score, we examined the specific correlation between each TME-infiltrating cell type and the MMP score using Spearman correlation analysis, which showed a strong correlation of the majority in Figure 3A. Our study showed that TME immune cell infiltration was significantly increased in tumour with high MMP scores, showing a significant positive correlation with follicular helper T cells, CD4 memory activated, naive B cells, and activated dendritic cells (Figure 3B, C).

Upon investigation of the correlation between the MMP score and human leukocyte antigen (HLA) related-molecules, we found that the MMP score showed a significant positive correlation with immune checkpoints (Figure 3D, Supplementary Figure 3 and 4). Among them, CD274 and ICOS showed the most significant positive correlation (Figure 3E, F). In addition, we found that different types of HLA were positively correlated with the MMP score (Figure 3G), thereinto, HLA-E and HLA-F being the most prominent (Figure 3H, I). This shows that the MMP score can positively regulate many immune checkpoints in EC, such as HLA molecules and interleukins. To investigate the role and functions of immune cells in EC, we examined the association between MMP scores and immune cells using linear regression analysis. Significant positive correlations were observed, such as CD4^+^ memory activation in T cells. Most immune checkpoints, such as CD276, TNFRSF9, CD274 and so on, were significantly different. For immune regulation, we found a correlation between the MMP score and interleukins (Figure 3J), thereinto TLSP being the most significant (Figure 3K). These results demonstrated that the MMP score directly positively regulates the immune function of T cells. Subsequently, we measured mRNA expression to further explore the relationship between the MMP score and stem-like properties of ECs tumour cells (Figure 3L). The MMP score was significantly negatively associated with stem cell mRNAs, indicating that a higher MMP score is closely correlated with lower tumour stem cell activity and a higher degree of tumour differentiation. All of the above results show that the MMP expression of EC play a significant role in immunologic function and immune infiltration.

### 3.4 Clinical features of the MMP expression patterns

**Figure 4:**
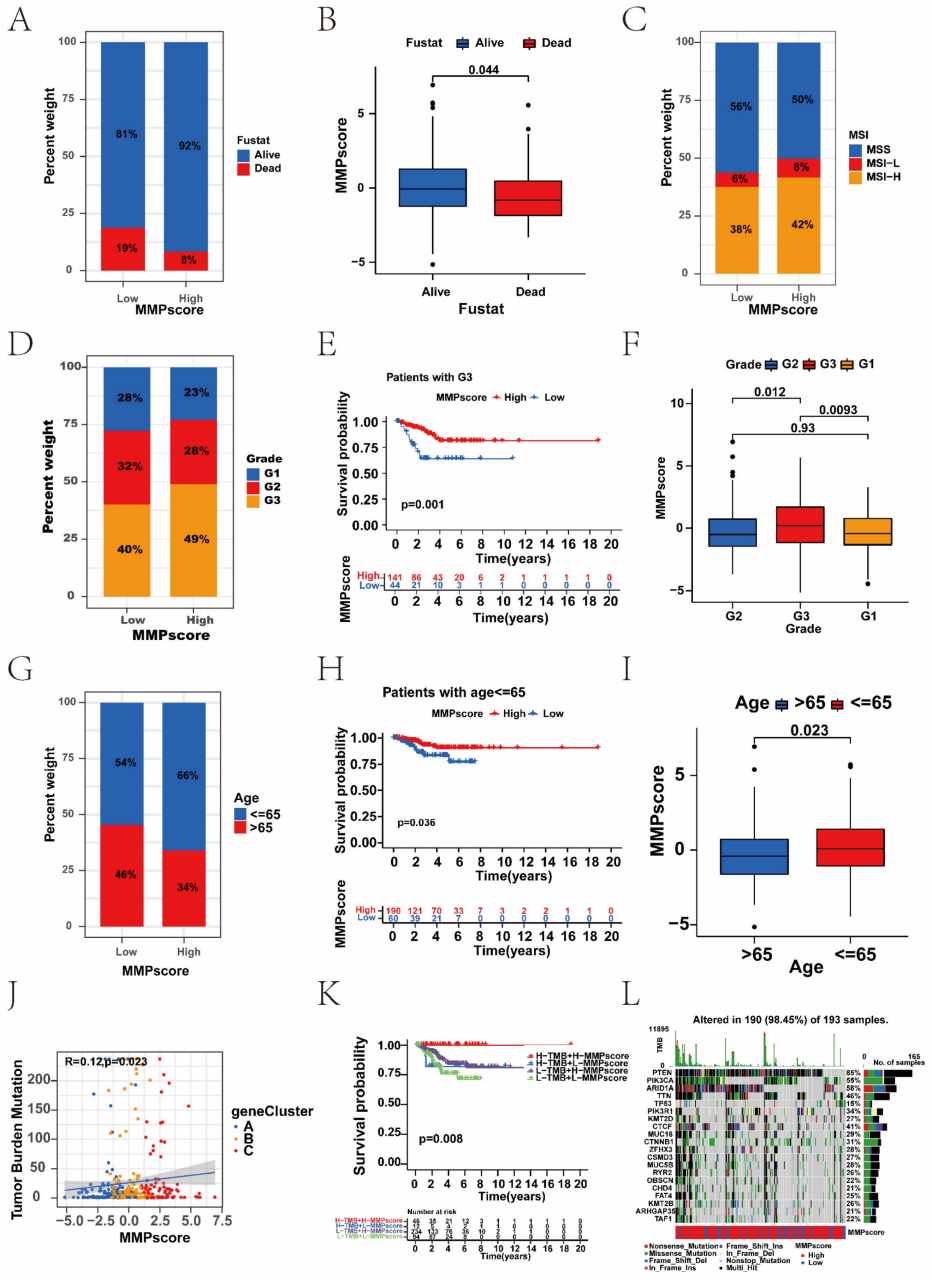
Correlation between clinicopathological characteristics and the MMP score. (A) The proportions of living and dead EC patients in the low and high MMP score groups. Blue, living patients; red, deceased patients. (B) MMP score based on survival status. Blue, living patients; red, deceased patients. (C) Relationships between MMP score and MSI. Blue, MSS group; red, MSI-L group; yellow, MSI-H group. (D) The proportions of patients with three clinical grades in the low and high MMP score groups. Blue, grade 1 group; red, grade 2 group; yellow, grade 3 group. (E) Kaplan-Meier survival analysis for high and low MMP score patient groups in the patients with G3. Log-rank test, *P* = 0.001. (F) Differences in the MMP score among distinct grade clinical response groups. (G) The proportion of patient ages in the low or high MMP score group. Blue, patients ≤65 years; red, patients >65 years. (H) Kaplan-Meier survival analysis for high and low MMP score patient groups in patients ≤65 years. Log-rank test, *P* = 0.036. (I) Differences in the MMP score among distinct age clinical response groups. (J) Correlation between the MMP score and tumour burden mutation in the MMP gene clusters. MMP gene cluster-A, blue; MMP gene cluster-B, yellow; MMP gene cluster-C, red. (K) Survival analyses stratified by both the MMP score and TMB using Kaplan-Meier curves (*P* = 0.008, log-rank test). Red, the patients with a high TMB and high MMP score; blue, the patients with a high TMB and low MMP score; purple, the patients with a low TMB and high MMP score; green, the patients with a low TMB and low MMP score. (L) Mutational landscape of genes in the TCGA UCEC cohort stratified by high versus low MMP score subgroups. Each column represents individual patients. The upper bar plot shows TMB, the right bar plot shows the mutation frequency of each gene in separate MMP score groups. MSS, Microsatellite-stable; MSI-L, Microsatellite instability-low; MSI-H, Microsatellite instability-high; TMB, tumour mutational burden.

We used the MMP score to systematically evaluate EC in terms of clinical characteristics including age, clinical stage, and fustat status. Based on these results, we performed a survival analysis to explore the distribution of survival status between patients with high and low MMP scores. In the low MMP score group, 81% of patients were alive and 19% were dead, and in the high MMP score group, 92% of patients were alive and 8% were dead (Figure 4A). It was showed significant differences (*P* = 0.044) between scores and survival status in Figure 4B. In addition, we analysed the correlation between MSI and MMP scores, found that patients in the high MMP score group had a higher proportion of MSI-H (Figure 4C). We observed an elevated proportion of patients with an advanced clinical grade in the high-scoring group (Figure 4D). Patients with G1 or G2 grade had lower MMP scores than those with G3 grade (Figure 4F). In addition, we also calculated the MMP score among patients of different ages and found a significant difference between patients <65 and ≥65 years. Thereinto, a high MMP score was also significantly correlated with the patients' age, especially in patients ≤65 with a better survival prognosis (Figure 4G, I). To further assess the prognostic value of the MMP score in the different subgroups, we performed Kaplan-Meier analyses (Figure 4E, H). We found that the MMP score exhibited prognostic power in various subgroups. Among women, those younger than 65 years, and those with G3 disease, the high MMP score group had a better prognosis than the low MMP score group. These results demonstrate that MMP score has the potential to act as a biomarker for assessing clinical characteristics and predicting prognosis in patients with EC. To better demonstrate the features of the MMP signature, we also verified the correlation between the TME-infiltrating cells and the MMP score, which showed a positive relationship. A positive correlation between the TMB and MMP scores in the three gene clusters is shown in Figure 4J (R = 0.12, *P* = 0. 023). We divided the patients with EC into two categories: L-TMB and H-TMB. Kaplan-Meier curves revealed that the H-TMB had the longer survival (*P* = 0.023) (Supplementary Figure 5A). Next, we evaluated the survival among EC patients with TMB and MMP scores. Based on previous results, we believed that patients with high MMP and TMB scores should have the most significant survival advantage (Figure 4K). We then analysed the distribution differences of somatic mutations between the low and high MMP score groups in the TCGA cohort (Figure 4L, and Supplementary Figure 5B).

Some studies have shown that patients with a higher TMB have sustained clinical benefits and survival. In our analysis, we reached a similar conclusion: the high MMP score group presented with more extensive tumour mutations than the low score group.

### 3.5 The role of the MMP score in predicting immunotherapeutic benefits

**Figure 5:**
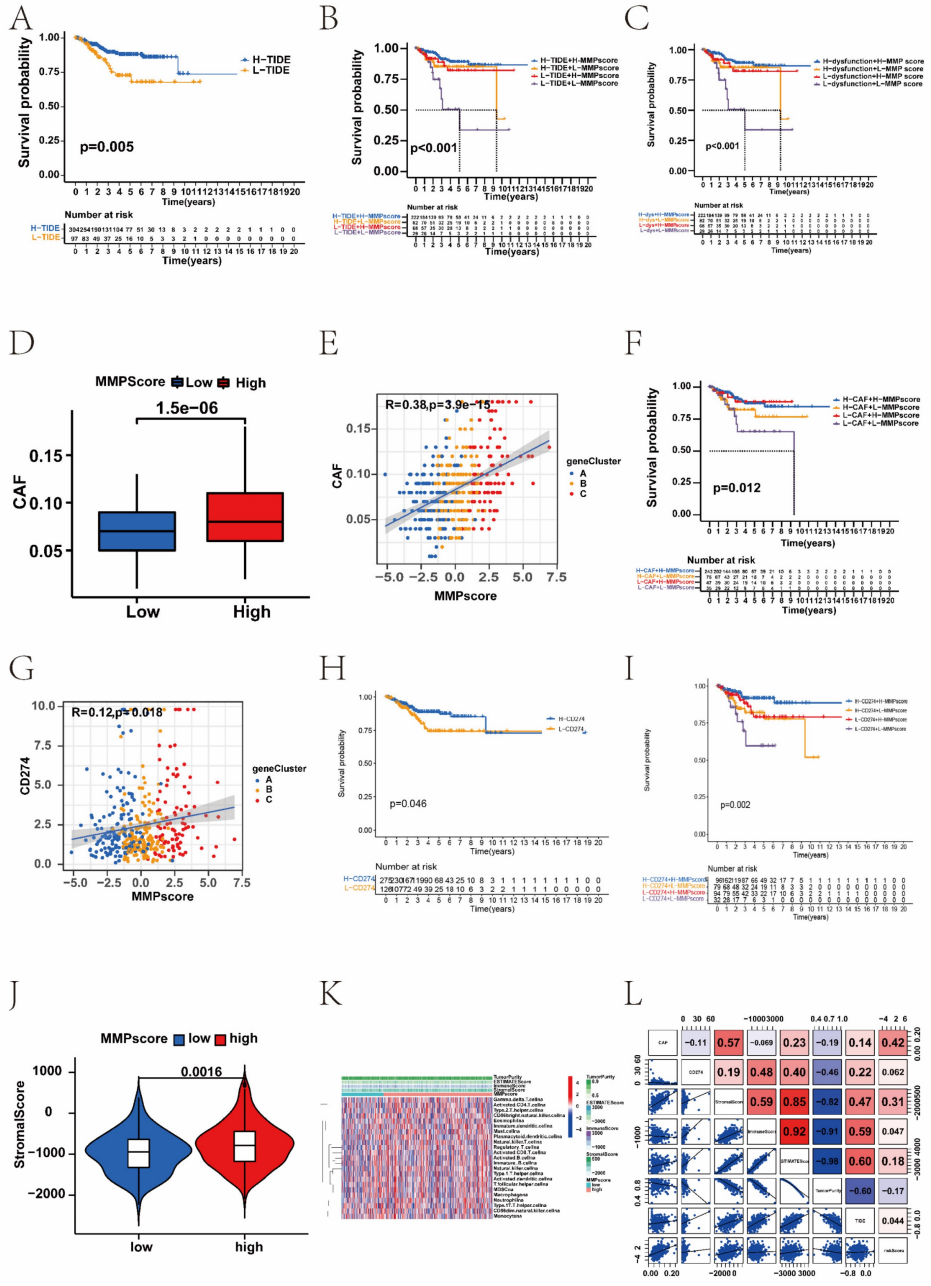
Association between the MMP score and immune. (A) Survival analysis for the patients with a high TIDE score and the patients with a low TIDE score (*P* = 0.005, Log-rank test). Blue, high TIDE group; yellow, low TIDE group. (B) Survival analyses stratified by both the MMP score and TIDE using Kaplan-Meier curves (*P* < 0.001, Log-rank test). (C) Survival analyses stratified by both the MMP score and dysfunction using Kaplan-Meier curves. (D) The relative distribution of CAF scores was compared between the low and high MMP score groups. There was a significant difference in the predicted response to immunotherapy between the two MMP score groups. (E) The correlations between CAF and MMP score in three gene clusters. (F) Survival analyses stratified by both the MMP score and CAF using Kaplan-Meier curves. (G) The correlations between CD274 and MMP score in three gene clusters. (I) Survival analyses stratified by both the MMP score and CD274 using Kaplan-Meier curves. (J) The violin plot shows the difference in the stromal score between the low and high MMP score groups. (K) GSVA enrichment analysis showing the activation states of immune functions and immune cells in distinct MMP score groups, and the tumour purity, ESTIMATE score, immune score, and stromal score were used as patient annotations. Red, activated state; blue, inhibition state. (L) The correlation analysis of MMP score and immune including CAF, CD27, StromalScore, ImmuneScore, STIMATEScore, TummorPurity, and TIDE. TIDE, Tumor Immune Dysfunction and Exclusion; CAF, Cancer-associated fibroblast.

We found that the TIDE score was also associated with prognosis; patients with the high TIDE score had a distinctly better prognosis than the low score group (Figure 5A). Survival analysis based on both TIDE and MMP scores showed that patients with low TIDE and MMP scores had the worst prognosis, whereas patients with high MMP and TIDE scores had the best prognosis (Figure 5B). We further analysed the targeted CAF, CD274, and immune dysfunction. Consistent with the TIDE score distribution, there was positive correlation between MMP scores and immune dysfunction, which patients with high immune dysfunction had more significant prognostic advantage than the low (Supplementary Figure 5C). Survival analysis combining immune dysfunction with the MMP score showed that patients with a high MMP score and immune dysfunction had the best survival (Figure 5C). Moreover, we found similar results in the analyses of CAF and CD274. Patients with a high MMP score were more likely to have CAF, and patients with a high CAF had a significant prognostic advantage over those with the low. Survival analysis combining CAF with MMP scores showed that patients with a high MMP score and high CAF had the best survival (Figure 5D-F). Patients with high MMP scores were more likely to express CD274, and patients with high CD274 expression had a significant prognostic advantage over those with low. Survival analysis combining CD274 with the MMP score showed that patients with a high MMP score and high CD274 had the best survival (Figure 5G-I). Therefore, regardless of TIDE, immune dysfunction, CAF, and CD274 scores, patients in the high MMP score group consistently had better survival than those in the low, indicating the value of the MMP score in predicting the therapeutic effect on ICB.

To explore the correlation between the MMP score and the TME, we analysed the stromal, immune, and ESTIMATE scores respectively (Supplementary Figure 5D). Patients with high MMP scores showed higher stromal scores than those with low (Figure 5J). Therefore, compared with the low MMP score group, the high score group had tumours with more abundant stromal components, which means they had stronger immune function and better prognosis. Furthermore, we performed GSVA enrichment analysis to compare the differences in the activation states of immune functions and immune cells between distinct MMP score groups. As shown in the heatmap, the high MMP score group showed significant enrichment in multiple immune pathways such as mast cells and regulatory T cells (Figure 5K). To assess the potential relationship between the MMP score and scores representing the seven functional states, we conducted a series of Pearson correlation analyses of the functional states (Supplementary Figure 5L). Specifically, we found that in EC, the MMP score was significantly positively correlated with CAF, CD274, the stromal score, immune score, STIMATE score, and TIDE, but negatively correlated with tumour purity.

### 3.6 The MMP score is predictive of the therapeutic response to chemotherapeutic drugs in EC patients

The efficacy of doxorubicin-based chemotherapy as a first-line therapy after EC surgery has been widely demonstrated. Researchers continue to investigate novel drugs for the treatment of EC. Considering the differences in survival and response to ICB in the different MMP score groups, we analysed the ability of the MMP score to predict the response of different chemotherapeutic drugs, including TG101348, WH-4-023, BMS-754807, Foretinib, AUY922 and BX-795 (Supplementary Figure 6A-F). These results indicated that EC patients with low MMP scores have a better therapeutic response to these drugs than patients with the high. We examined the correlation between the MMP score and sensitivity to these drugs. Significant positive correlations were observed in TG101348, WH-4-023, and BMS-754807 respectively (Supplementary Figure 6G-L). These results suggested that our prognostic model was an essential indicator for EC patients to choose antitumour drugs. We found a series of expression levels of MMPs in EC patients from the website (http://www.proteinatlas.org/), The immunohistochemical staining results showed that *MMP-9, MMP-11, MMP-15* and *MMP-24* were all highly expressed in EC tissue (Supplementary Figure 8). These results were consistent with those of our study above, indicating that patients in the high MMP score group showed a better prognosis, but patients with low MMP score could have a poor prognosis.

### 3.7 Single-cell RNA-seq reveals extensive heterogeneity of MMPs in EC

To understand the cellular diversity and molecular features of the endometrial tissue in EC patients, a normal and an EC sample were collected for single-cell RNA-seq data.

**Figure 6:**
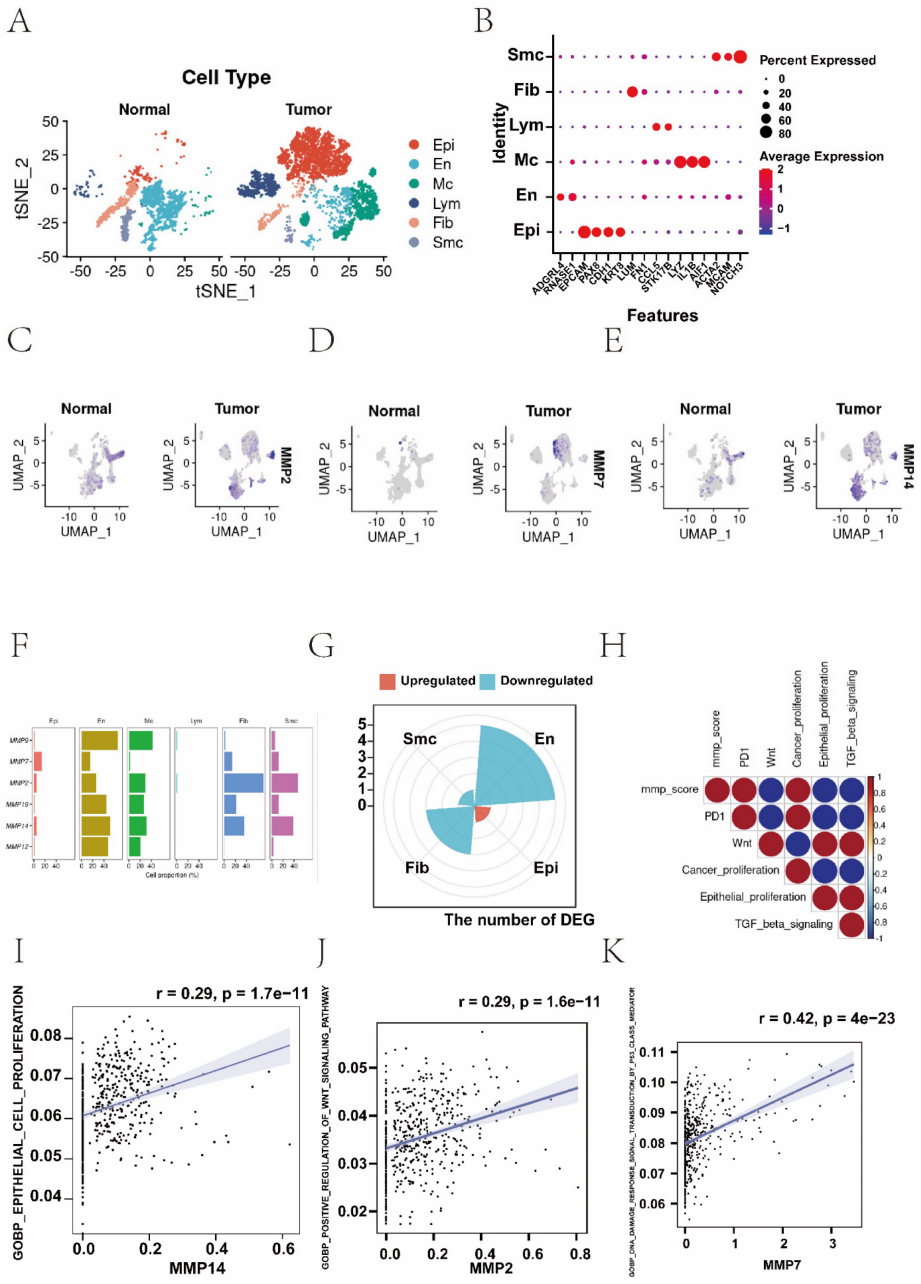
(A) The t-SNE plot demonstrating main cell types in normal and EC samples. (B) Expression patterns of canonical specific markers in each cell type. Each dot represents a gene, of which the colour saturation indicates the average expression level, and the size indicates the percentage of cells expressing the gene. (C-E) The t-SNE plot shows the expression levels of *MMP-14*,* MMP-7*, *MMP-2* in normal and EC samples, respectively. (F) Percentage of MMPs gene in each cell type. (G) The distribution of up- and down-regulated genes of DEG in each cell type. (H) Correlations between MMP score and the classical biological gene signatures in EC using Spearman analysis. The negative correlation was marked with blue and positive correlation with red. (I) Correlation between the *MMP-14* and epithelial cells proliferation (r=0.29, *P* < 0.001, Spearman correlation analysis). (J) Correlation between the *MMP-2* and WNT signalling pathway (r = 0.29, *P* < 0.001, Spearman correlation analysis). (K) Correlation between the *MMP-7* and P53 mediated pathway (r = 0.42, *P* < 0.001, Spearman correlation analysis). t-SNE, t-distributed stochastic neighbour embedding.

After quality control, 9012 cells were retained for subsequent analysis, comprising 2923 cells from normal sample and 6089 from EC sample. Six known cell types including 3096 epithelial cells, 2161 endothelial cells, 1543 macrophages, 959 lymphocytes, 839 fibroblasts, 414 smooth muscle cells, were identified and annotated by using classical marker genes (Figure 6A and B, Supplementary Figure 7A). The expressions of *MMP-14* and *MMP-7* were up-regulated in EC tissue, but *MMP-2* was down-regulated in EC tissue (Figure 6C-E, Supplementary Figure 7C). To explore the distribution ratio of MMPs in each cell (Supplementary Figure 7B), it can be seen that the proportion of MMPs in endothelial cells is the highest, followed by macrophages (Figure 6F). Most up-regulated DEGs were clustered in epithelial cells, while the down-regulated DEGs were mostly clustered in other cell types (Figure 6G). Furthermore, to probe the association between the MMP regulators and progression of EC, we used AUCell and GSEA database to perform the correlation between MMP score and classical pathways in EC to explore the influence of MMP regulators on cancer-related pathways. Notably, we found MMP score were upregulating PD-1 (R > 0.8) and cancer proliferation (R > 0.8), but downregulating carcinogenic activation pathways, such as Wnt pathway (R < -0.8), epithelial cell proliferation (R < -0.8) and TGF-β pathway (R < -0.8) in EC patients (Figure 6H). Additionally, we analysed the relationship between individual MMP genes and the classical biological gene pathways. The results showed *MMP-14* and epithelial cells proliferation (r = 0.29, *P* < 0.001), *MMP-2* and WNT signalling pathway (r = 0.29, *P* < 0.001), *MMP-7* and P53 mediated pathway (r = 0.42, *P* < 0.001), *MMP-7* and abnormality of complement system (r = 0.36, *P* < 0.001) were positively correlated, respectively (Figure 6I-K, Supplementary Figure 7D). In summary, there was significant heterogeneity in the expression of MMPs in single-cell analysis.

### 3.8 MMP expression is generally increased in EC tissue

We verified the expression of the MMP gene set in EC tissue using immunohistochemistry, and found that MMP expression in normal endometrial tissue showed high expression, demonstrating the effectiveness of the MMP score (Supplementary Table 1). To verify whether the expression of the MMP gene set is generalized at the molecular level in tissue of patients with EC, we conducted HE and qRT-PCR experiments on endometrial tissue from three cases of EC and three healthy individuals. HE staining revealed cell polarity disorder, increased mitotic figures, and abnormal cells breaking through the basal layer in EC tissue, which is consistent with the diagnosis of EC.

**Figure 7:**
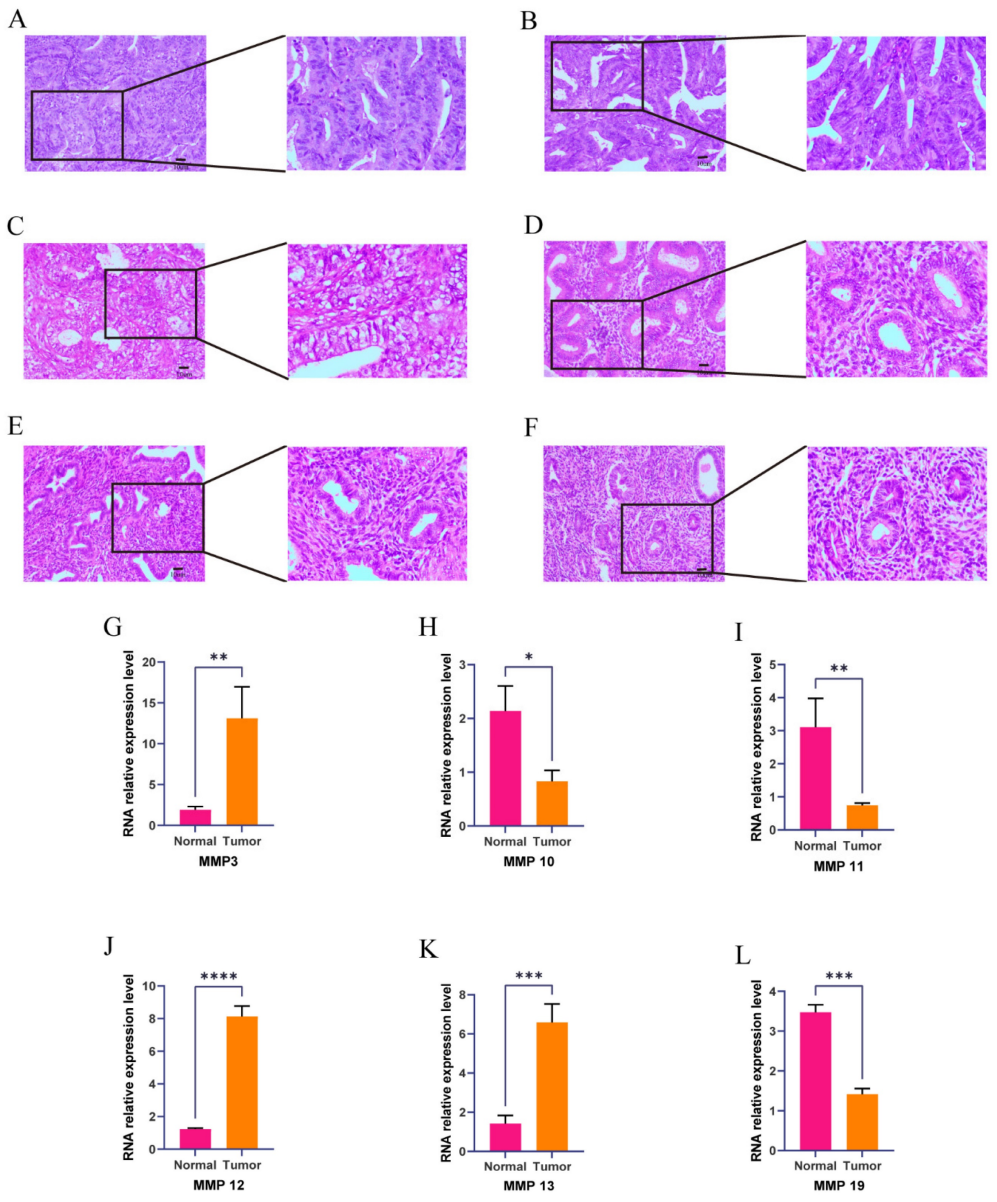
(A-F) Hematoxylin and eosin (H&E) staining was performed to observe pathological changes of endometrial tissue in normal and EC group. (A, B, C, normal tissue; D, E, F, EC tissue; magnification: 100x, scale bar = 10µm). (G-L) Expression levels of mRNA of MMPs in EC and control. The mRNA expression levels of *MMP-3*, *MMP-10*, *MMP-11*,* MMP-12*, *MMP-13*, and *MMP-19* in patients with EC or controls were measured by RT-qPCR. GAPDH were used as a loading control. Data are based on the mean ± SD of triplicate independent experiments. *P* values were obtained using Student's t test. (EC, n = 3; Controls, n = 3; *, *P* < 0.05; **, *P* < 0.01; ***, *P* < 0.001; ns, nonsignificant).

HE staining of endometrial tissue in the control group conformed with normal endometrial tissue pathological characteristics (Figure 7A-C). We observed that the expression of* MMP-3*, *MMP-12* and *MMP-13* genes in EC tissue was generally higher than that in normal endometrial tissue (Figure 7D-F). QRT-PCR analysis showed that the mRNA expression of* MMP-3*,* MMP-12* and *MMP-13* in EC tissue was significantly greater than the normal group (*P* = 0.0074, *P* < 0.001, and *P* = 0.001, respectively). mRNA expression levels of *MMP-10*, *MMP-11*, and *MMP-19* in the normal endometrial tissue was greater than the EC group (*P* = 0.0113, *P* = 0.0095, *P* = 0.0001, respectively) (Figure 7G-L). These results showed there were changes in expression levels of MMPs during the process from normal to cancerous, which further verified that MMPs can act as cancer-related genes to regulate the occurrence and development of EC.

## 4. Discussion

Currently, 23 MMPs are identified in humans. Based on the specificity of substrate and structure, MMPs can be classified into collagenases, gelatinases, stromelysins, matrilysins, metalloelastases, enamelysins, membrane-types, and so on 27. They can break down the basement membrane, bind to the surface of cancer cell, facilitate ECM remodelling and release membrane-bound growth factors in TME, which above eventually ultimately lead the invasion and metastasis of tumours 28, 29. Among them, *MMP-9* is the most extensively researched. Li *et al.* pointed out that *MMP-9*, as a gelatinase, could degrade gelatin, collagen and elastin through proteolytic cleavage to promote ECM remodeling, therefore, its overexpression might be a useful predictor of poor prognosis of EC 30. *MMP-7* can predict a more aggressive phenotype of colon cancer and is inversely correlated with patient survival 31.

*MMP-11* is a potential tumour marker and therapeutic target for advanced prostate cancer 32. Levels of *MMP-7* positively correlate with GC invasion, lymph node metastasis, peritoneal dissemination, and patient survival 33-35. An increasing evidence show that the expression of MMPs is related to the progression of gynaecological malignancies 36-38. Luis *et al.* found that tumour budding count, which is connected to tumour migration in the context of EMT, was regulated by MMPs in breast cancer patients 39. However, the influence for the MMP regulators has not been studied on EC diagnosis and prognosis, and the mechanism underlying the role of MMPs in EC warrants further investigation.

Accumulating evidence indicates that the expression profile plays an indispensable role in inflammation, immunity, and inhibition of tumour progression 7, especially in the development and progression of digestive tract tumours 9, 40, 41; however, there are no related studies on EC. Furthermore, most studies have focused on a single TME cell type or single protease, but the overall TME infiltration characteristics mediated by the combined effects of multiple MMPs have not been comprehensively recognized 4. In this study, we analysed the clinical information and transcriptome data of EC patients from TCGA, and identified three distinct MMP expression patterns which displayed differences in immune cell infiltration and different disease prognosis. In addition, GSVA enrichment analysis revealed multiple tumour-associated signalling pathways enriched in MMP cluster B. To quantify the MMP expression pattern in individual EC patients, we established a scoring system based on the expression of MMP regulatory factors, and EC patients were divided into high and low MMP score groups. High MMP scores had a better prognosis, along with more abundant immune cell infiltration and stronger antitumour immunity. Although low MMP score had a worse prognosis than high, they responded better to immunotherapy. Our work indicated that MMP score could be a potential tool to evaluate MMP expression patterns, immune cell infiltration, response to pharmacotherapy, clinicopathological features, and survival outcomes in EC, and has the potential to provide novel areas for the study of epigenetics in EC. Stroma can be confined to the tumour envelope or penetrate the tumour itself, making immune cells appear truly inside the tumour. More importantly, we found that MMP cluster A exhibited a distinct stromal activation status, combined with TME cell infiltration features in each cluster, and patient prognosis was the opposite of what we expected; therefore, we speculated that stromal activation in MMP cluster A inhibited the antitumour effect of immune cells. Significant prognostic differences were observed between the two clusters, confirming the reliability of our immunophenotypic classification of different MMP expression patterns. Therefore, by fully exploring the characterisation of TME cellular infiltration induced by different MMP expression patterns, it was demonstrated that MMP cluster A could further lead to a poor prognosis through the function of suppressed immune cells. In addition, the results showed that the genetic and expression alterations of MMPs between EC tissue and normal tissue had a certain heterogeneity, indicating that MMP expression imbalance may play an important role in the accuracy and progression of EC. Our seminal exploration of the role of the overall MMP expression pattern in the infiltration of TME cells will contribute to a deeper understanding of the mechanism of the TME antitumour immune response and a more effective strategy for guiding immunotherapy.

In this study, similar to the clustering results for MMPs expression, three genomic subtypes were identified that were significantly associated with matrix activation and immune responses. This again demonstrated that MMP expression had important implications in shaping different TME. Therefore, a comprehensive evaluation of MMP expression patterns would enhance our understanding of TME cellular infiltration. However, previous analyses were mainly based on patient populations and could not accurately predict the expression pattern in individual patients; therefore, considering the individual heterogeneity of MMP expression, its pattern must be urgently quantified in single tumours. In this study, this deficiency was compensated by constructing an MMP scoring system, evaluating MMP expression patterns and visualising property changes in individual patients. The expression pattern, which was dominated by the MMP cluster B expression signature, exhibited a high MMP score, suggesting that the MMP score is a reliable and powerful tool to comprehensively assess the expression pattern of MMPs in individual tumours and can be used to further determine the TME infiltration pattern, namely, the tumour immunophenotype. More importantly, the MMP score showed good assessment ability in terms of patient clinical characteristics, including the tumour differentiation level, mutation burden, pathological stage, age, and clinical prognosis, and could guide clinical treatment. A comprehensive analysis showed that the MMP score is an effective indicator of biological prognosis in endometrial cancer. Our MMP score showed excellent predictive power for precision endometrial cancer immunotherapy, utilizing the features of immune escape.

Our data revealed a significant correlation between the MMP score and TMB, which was patients with a combination of high MMP score and high TMB showed a great survival advantage. Consistent with previous studies, the increased release of MMPs, as well as their cell membrane expression, would lead to a breakdown of the ECM and favour infiltration 42, 43. Moreover, our study found that MMP expression was associated with shaping different stromal and carcinogenic activation pathways, such as TGF-β pathway components and Wnt pathway. Previous studies confirmed that the TGF-β signalling can bind TGF-β and *TGFBR2* to inhibit EC migration through the phosphorylation 44. Wnt pathway is well-known to play a vital role in multiple cellular functions, such as embryonic development, cell proliferation, adult tissue homeostasis, and so on. In particular, aberrant activation of Wnt/β-catenin pathway correlates with tumourigenesis of EC, including accelerating proliferation of EC cells 45. In this work, we showed MMP genes played a non-negligible role in shaping different stromal and immune TME landscape, implying MMPs could affect the therapeutic efficacy of ICB. The MMP gene signature with integrated various biomarkers including TMB, TIDE, CAF and CD274, could be the more effective predictive strategy for immunotherapy. In addition, the correlation between MMP score, tumour stage, and prognosis analysis showed a significant effect for all grades. The correlation of the MMP score with patient fustat and age factors, and its predictive effect on survival were also obvious.

Pan-cancer analysis has shown that MMPs have a prognostic value only in clear-cell renal cancer 46-48. This study elucidated the role of MMPs in cancer by developing an MMP scoring system that may serve as an independent marker for predicting patient survival and prognosis and provide new insights into ECs immunotherapy. These new ideas may target MMP-related genes, reverse unfavourable TME cell infiltration, and help develop novel drug combination strategies or immunotherapeutic agents in the future. We further investigated the relationship between MMP score and pharmacotherapy response. MMP score was significantly correlated with predictors of the immune response, such as the TIDE score, indicating that MMP expression affects the therapeutic effect of immunotherapy and can be used to improve the personalised treatment of EC patients. Additionally, a higher MMP score was significantly related to higher TIDE and immune dysfunction scores. Patients with a higher TIDE score tended to have a stronger immune dysfunction score and a decreased ability to kill cancer cells, which could explain why they had a worse response rate to ICB. Conversely, although patients with low MMP scores had lower TIDE scores and worse prognoses, they were more likely to benefit from ICB treatment. Therefore, patients with low MMP scores may have prolonged survival after ICB therapy. Moreover, the MMP score can predict response to pharmacotherapy. These results suggest that the MMP score can be used to develop individualised treatment plans for patients with EC. Overall, we provide new ideas for improving patient clinical responses to immunotherapy, identifying distinct tumour immune phenotypes, and promoting personalised EC immunotherapy in the future.

## 5. Conclusions

In summary, this study demonstrated a broad regulatory mechanism of the EC TME via the MMP expression landscape. Differences in the MMP expression patterns are non-negligible factors that contribute to the heterogeneity and complexity of individual TME. The MMP score can be used in clinical practice to comprehensively evaluate the MMP expression patterns of individual patients and their corresponding TME cell infiltration characteristics, further determine the tumour immune phenotype, and guide more effective clinical practice. Moreover, the MMP score exhibited a strong predictive function in EC patient survival analysis, which could provide guidance for clinical workup. A comprehensive assessment of the MMP expression patterns in individual tumours will enhance our understanding of the characteristics of cellular infiltration into the TME. The correlation between MMP score, immune checkpoints, and immune cells may provide strategies and directions for subsequent immunotherapy research.

## Supplementary Material

Supplementary figures and table.

## Abbreviations

MMPs: Matrix metalloproteinases
EC: Endometrial cancer
TME: Tumour microenvironment
PCA: Principal component analysis
ROC: Receiver operating characteristic
scRNA-seq: single-cell RNA sequencing
GO: Gene Ontology
DEGs: Differentially expressed genes
ICB: Immunological checkpoint blockade
PMB: Postmenopausal bleeding
CAF: Cancer-associated fibroblast
ECM: Extracellular matrix
TCGA: The Cancer Genome Atlas
ICGC: International Cancer Genome Consortium
TCGA-UCEC: The Cancer Genome Atlas-Uterine Corpus Endometrial Carcinoma
UCEC-US: Uterine Corpus Endometrial Carcinoma- TCGA, US
GSVA: Geneset variation analysis
KEGG: Kyoto Encyclopedia of Genes and Genomes
ssGSEA: Single-sample gene set enrichment analysis
TIDE: Tumor Immune Dysfunction and Exclusion
MSS: Microsatellite-stable
MSI-L: Microsatellite instability-low
MSI-H: Microsatellite instability-high
TMB: tumour mutational burden
qRT-PCR: Quantitative real-time polymerase chain reaction
HR: Hazard ratios
KEGG: Kyoto Encyclopedia of Genes and Genomes
HLA: Human leukocyte antigen
