# Supplementary material for: Development and validation of matrix metalloproteinase for predicting prognosis and tumour microenvironment immune profiles in uterine corpus endometrial carcinoma
Source: J Cancer. 2024 May 30;15(12):4020–39. doi: 10.7150/jca.91277 (PMC11190773; doi:10.7150/jca.91277)
Supplement: Supplementary file 1 — Supplementary figures and table. [file jcav15p4020s1.pdf]

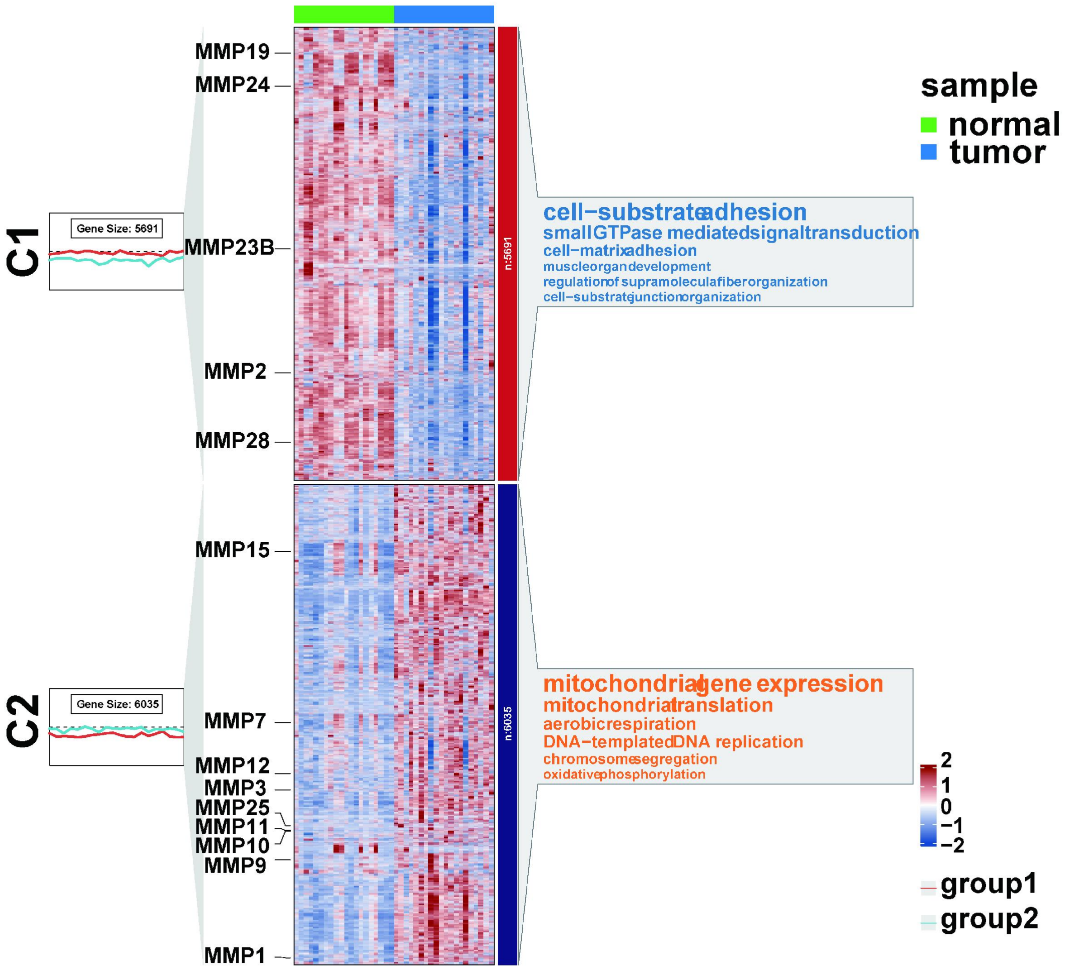

sample
normal
tumor
C1
Gene Size: 5691
MMP19
MMP24
MMP23B
MMP2
MMP28
n:5691
cell−substrate adhesion
small GTPase mediated signal transduction
cell−matrix adhesion
muscle organ development
regulation of supramolecular fiber organization
cell−substrate junction organization
C2
Gene Size: 6035
MMP15
MMP7
MMP12
MMP3
MMP25
MMP11
MMP10
MMP9
MMP1
n:6035
mitochondrial gene expression
mitochondrial translation
aerobic respiration
DNA−templated DNA replication
chromosome segregation
oxidative phosphorylation
2
1
0
−1
−2
group1
group2

Supplementary Figure 1. Expression patterns of MMPs in EC patients and healthy individuals. Heatmap of differentially expressed genes between EC and control groups; the MMPs in each module were annotated; the line graph showed the trend in the gene module expression, the text on the right showed the enriched pathways for each module gene. MMPs, matrix metalloproteinases;.

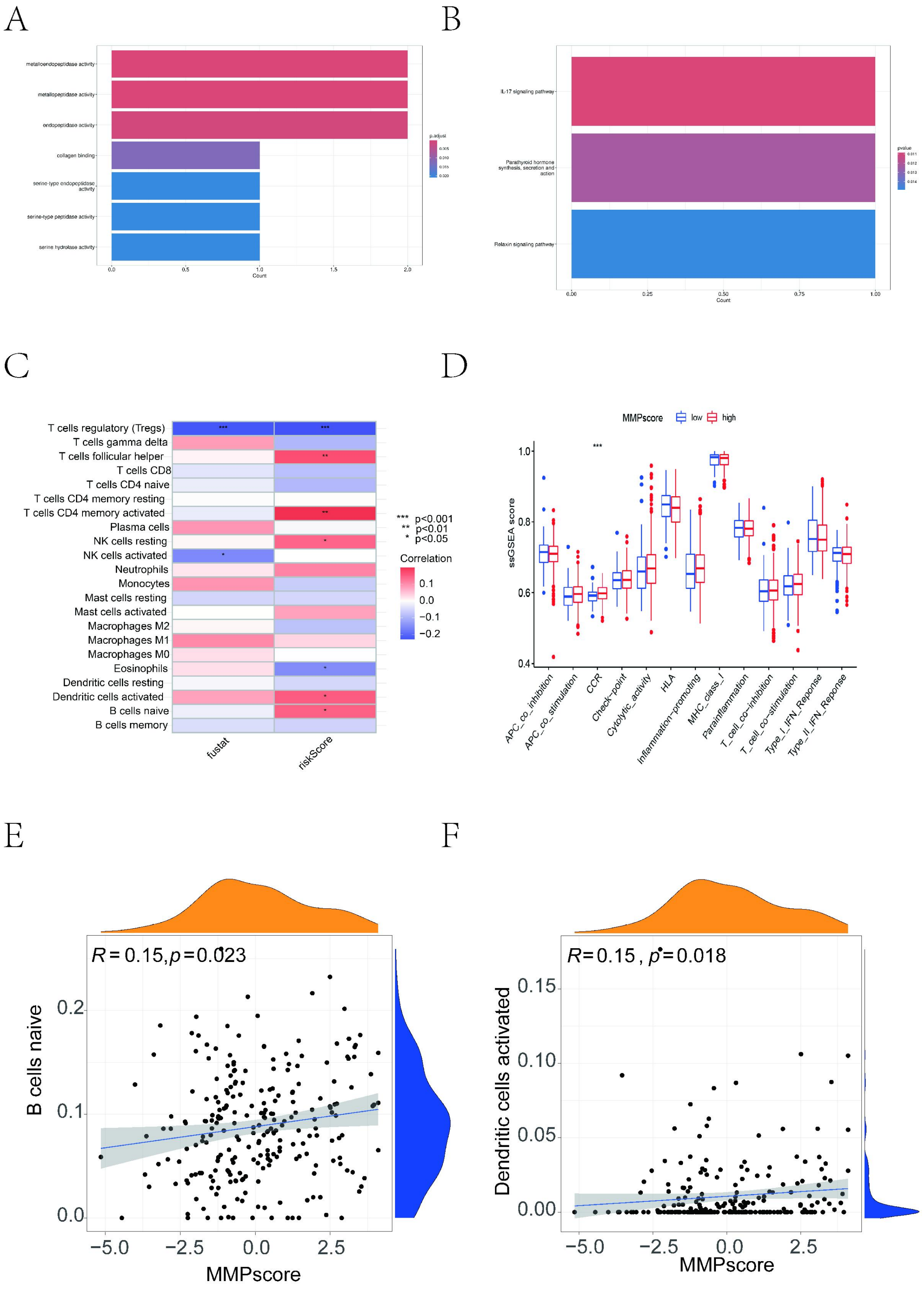
A
metalloendopeptidase activity
metallopeptidase activity
endopeptidase activity
collagen binding
serine-type endopeptidase activity
serine-type peptidase activity
serine hydrolase activity
0.0
0.5
1.0
1.5
2.0
Count
p.adjust
B
IL-17 signaling pathway
Parathyroid hormone synthesis, secretion and action
Relaxin signaling pathway
0.00
0.25
0.50
0.75
1.00
Count
pvalue
C
T cells regulatory (Tregs)
T cells gamma delta
T cells follicular helper
T cells CD8
T cells CD4 naive
T cells CD4 memory resting
T cells CD4 memory activated
Plasma cells
NK cells resting
NK cells activated
Neutrophils
Monocytes
Mast cells resting
Mast cells activated
Macrophages M2
Macrophages M1
Macrophages M0
Eosinophils
Dendritic cells resting
Dendritic cells activated
B cells naive
B cells memory
fustat
riskScore
*** p<0.001
** p<0.01
* p<0.05
Correlation
0.1
0.0
−0.1
−0.2
D
MMPscore
low
high
ssGSEA score
1.0
0.8
0.6
0.4
***
APC_co_inhibition
APC_co_stimulation
CCR
Check-point
Cytolytic_activity
HLA
Inflammation-promoting
MHC_class_I
Parainflammation
T_cell_co-inhibition
T_cell_co-stimulation
Type_I_IFN_Reponse
Type_II_IFN_Reponse
E
R = 0.15, p = 0.023
B cells naive
0.0
0.1
0.2
−5.0
−2.5
0.0
2.5
MMPscore
F
R = 0.15 , p = 0.018
Dendritic cells activated
0.00
0.05
0.10
0.15
−5.0
−2.5
0.0
2.5
MMPscore

Supplementary Figure 2. (A) CNV for MMPs genes using GO enrichment analysis. (B) CNV for MMPs genes using KEGG enrichment analysis. (C) Heatmap depicting the correlation between the ssGSEA score of 22 immune cells and survival. The colour depth of the squares represents the strength of the correlation. (D) The immune function scores were significantly different between the high and low MMP score groups. (E) Correlation between the MMP score and B cells native in endometrial cancer. R = 0.15, P = 0.023. (F) Correlation between the MMP score and dendritic activated in endometrial cancer. R = 0.15, P = 0.018. GO, gene ontology. KEGG, Kyoto Encyclopedia of Genes and Genomes; ssGSEA, single-sample gene set enrichment analysis.

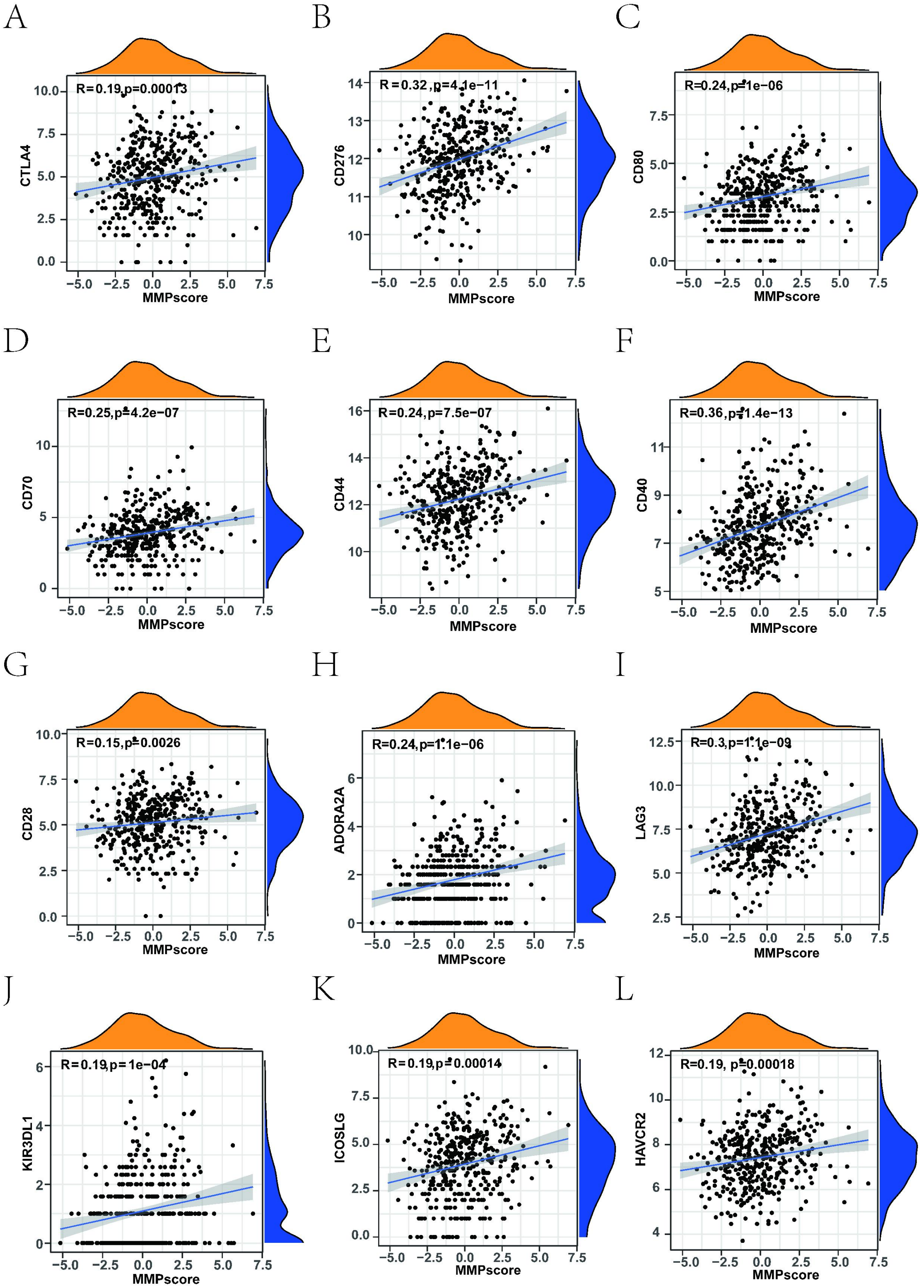
A
R=0.19,p=0.00013
CTLA4
MMPscore
B
R=0.32,p=4.1e−11
CD276
MMPscore
C
R=0.24,p=1e−06
CD80
MMPscore
D
R=0.25,p=4.2e−07
CD70
MMPscore
E
R=0.24,p=7.5e−07
CD44
MMPscore
F
R=0.36,p=1.4e−13
CD40
MMPscore
G
R=0.15,p=0.0026
CD28
MMPscore
H
R=0.24,p=1.1e−06
ADORA2A
MMPscore
I
R=0.3,p=1.1e−09
LAG3
MMPscore
J
R=0.19,p=1e−04
KIR3DL1
MMPscore
K
R=0.19,p=0.00014
ICOSLG
MMPscore
L
R=0.19, p=0.00018
HAVCR2
MMPscore

Supplementary Figure 3. (A) MMP score and CTLA4; (B) MMP score and CD276; (C) MMP score and CD80; (D) MMP score and CD70; (E) MMP score and CD44; (F) MMP score and CD40; (G) MMP score and CD28; (H) MMP score and ADORA2A; (I) MMP score and LAG3; (J) MMP score and LR3DL1; (K) MMP score and ICOSLG; (L) MMP score and HAVCR2.

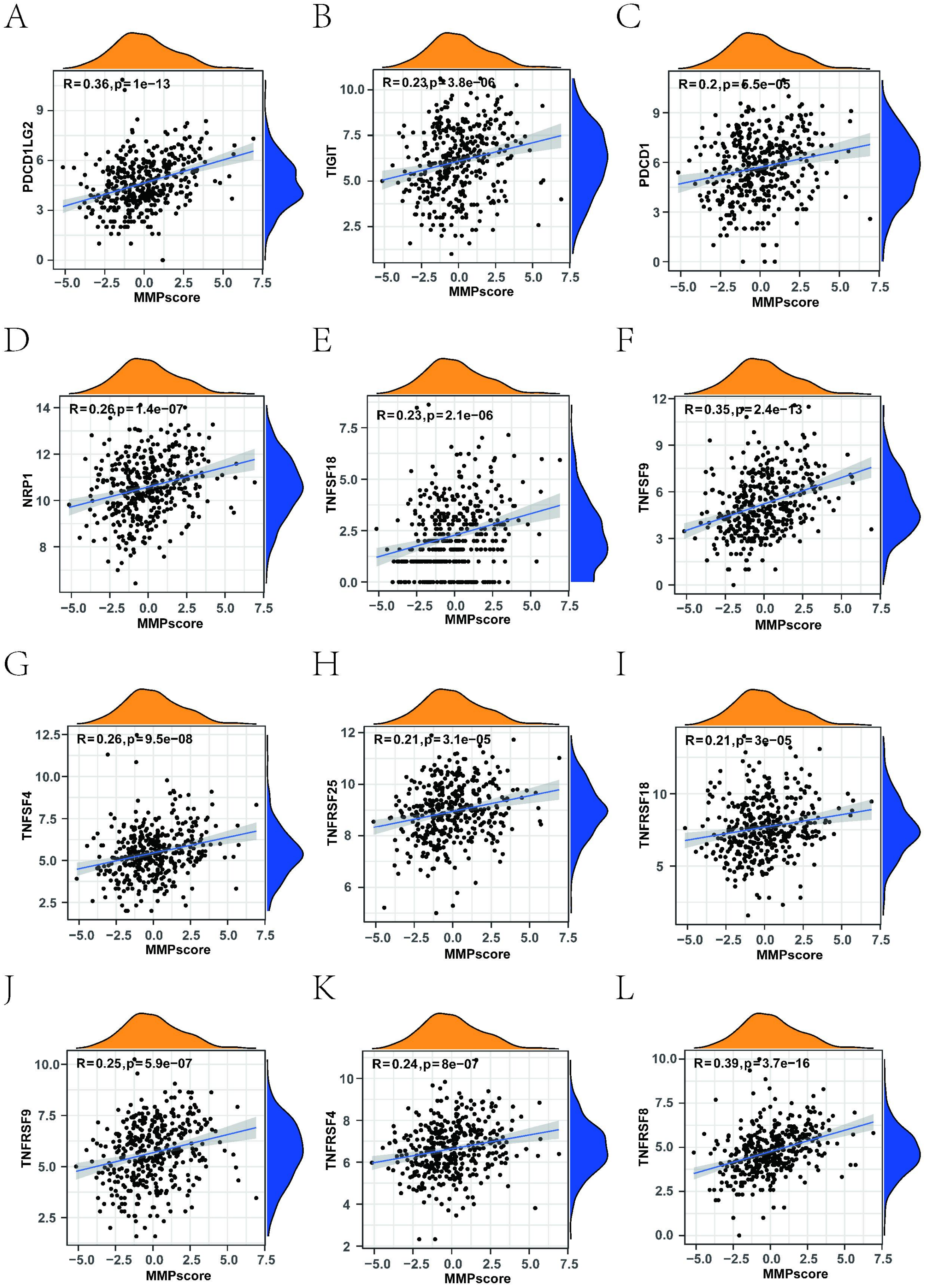

A
R=0.36,p=1e−13
PDCD1LG2
MMPscore
B
R=0.23,p=3.8e−06
TIGIT
MMPscore
C
R=0.2,p=6.5e−05
PDCD1
MMPscore
D
R=0.26,p=1.4e−07
NRP1
MMPscore
E
R=0.23,p=2.1e−06
TNFSF18
MMPscore
F
R=0.35,p=2.4e−13
TNFSF9
MMPscore
G
R=0.26,p=9.5e−08
TNFSF4
MMPscore
H
R=0.21,p=3.1e−05
TNFRSF25
MMPscore
I
R=0.21,p=3e−05
TNFRSF18
MMPscore
J
R=0.25,p=5.9e−07
TNFRSF9
MMPscore
K
R=0.24,p=8e−07
TNFRSF4
MMPscore
L
R=0.39,p=3.7e−16
TNFRSF8
MMPscore

Supplementary Figure 4. Correlation between the MMP score and immune checkpoints. (A) MMP score and PDCD1LG2; (B) MMP score and TIGIT; (C) MMP score and PDCD1; (D) MMP score and NRP1; (E) MMP score and TNFSF18; (F) MMP score and TNFSF9; (G) MMP score and TNFSF4; (H) MMP score and TNFRSF25; (I) MMP score and TNFRSF18; (J) MMP score and TNFRSF9; (F) MMP score and TNFRSF4; (G) MMP score and TNFRSF8.

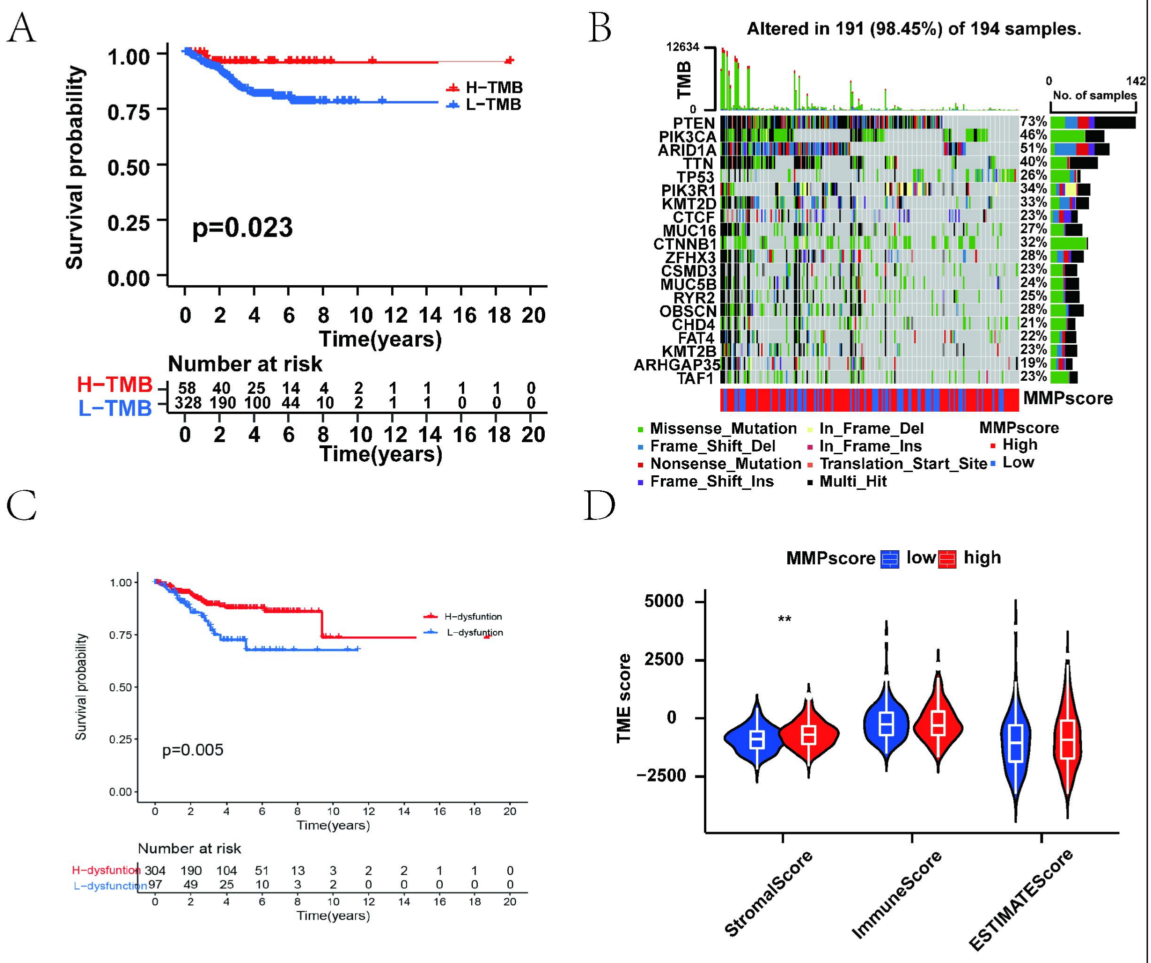
A
Survival probability
1.00
0.75
0.50
0.25
0.00
p=0.023
H−TMB
L−TMB
Time(years)
Number at risk
H−TMB 58 40 25 14 4 2 1 1 1 1 0
L−TMB 328 190 100 44 10 2 1 1 0 0 0
0 2 4 6 8 10 12 14 16 18 20
Time(years)
B
Altered in 191 (98.45%) of 194 samples.
TMB
12634
0
142
No. of samples
PTEN 73%
PIK3CA 46%
ARID1A 51%
TTN 40%
TP53 26%
PIK3R1 34%
KMT2D 33%
CTCF 23%
MUC16 27%
CTNNB1 32%
ZFHX3 28%
CSMD3 23%
MUC5B 24%
RYR2 25%
OBSCN 28%
CHD4 21%
FAT4 22%
KMT2B 23%
ARHGAP35 19%
TAF1 23%
MMPscore
Missense_Mutation
Frame_Shift_Del
Nonsense_Mutation
Frame_Shift_Ins
In_Frame_Del
In_Frame_Ins
Translation_Start_Site
Multi_Hit
MMPscore
High
Low
C
Survival probability
p=0.005
H-dysfuntion
L-dysfuntion
Time(years)
Number at risk
H-dysfuntion 304 190 104 51 13 3 2 2 1 1 0
L-dysfunction 97 49 25 10 3 2 0 0 0 0 0
0 2 4 6 8 10 12 14 16 18 20
Time(years)
D
MMPscore low high
TME score
5000
2500
0
−2500
**
StromalScore
ImmuneScore
ESTIMATEScore

Supplementary Figure 5. (A) Survival analyses of patients with a high-tumour mutational burden and low-tumour mutational burden using Kaplan–Meier curves (*P* = 0.023, Log-rank test). (B) Mutational landscape of genes in the TCGA UCEC cohort stratified by high versus low MMP score subgroups. Each column represents individual patients. The upper bar plot shows TMB, the right bar plot shows the mutation frequency of each gene in separate MMP score groups. (C) Survival analysis of the patients with a high dysfunction score and the patients with a low dysfunction score using Kaplan–Meier curves (*P* = 0.005, Log-rank test). Red, high dysfunction group; blue, low dysfunction group. (D) Analysis of the stromal, immune, and ESTIMATE scores between the low and high MMP score groups based on the ESTIMATE algorithm. Blue, low MMP score group; red, high MMP score group. TMB, tumour mutational burden

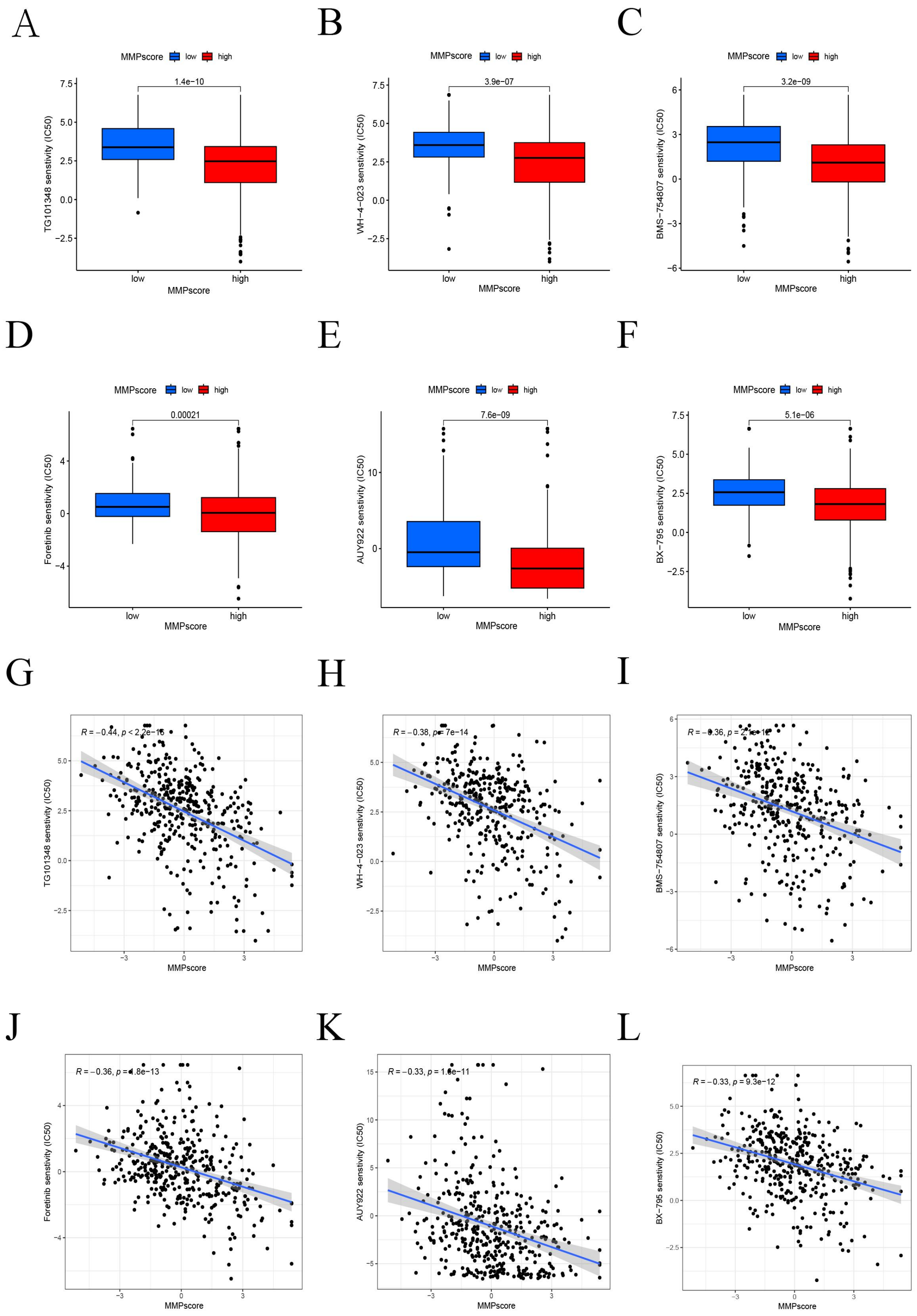
A
B
C
D
E
F
G
H
I
J
K
L
MMPscore low high
1.4e−10
TG101348 sensitivity (IC50)
3.9e−07
WH−4−023 sensitivity (IC50)
3.2e−09
BMS−754807 sensitivity (IC50)
0.00021
Foretinib sensitivity (IC50)
7.6e−09
AUY922 sensitivity (IC50)
5.1e−06
BX−795 sensitivity (IC50)
low
high
MMPscore
R = −0.44, p < 2.2e−16
R = −0.38
R = −0.36
R = −0.36, p = 4.8e−13
R = −0.33, p = 1.6e−11
R = −0.33, p = 9.3e−12

Supplementary Figure 6. Association between the MMP score and response to pharmacotherapy. (A–F) There was a significance difference in drug sensitivity between the two MMP score groups. (G–L) The correlation between the high and low expression of MMP score and the semi-inhibited concentration sensitivity of various drugs. Drugs: TG101348 sensitivity, WH-4-023 sensitivity, BMS-754807 sensitivity, Foretinib sensitivity, AUY922 sensitivity, and BX-795 sensitivity.

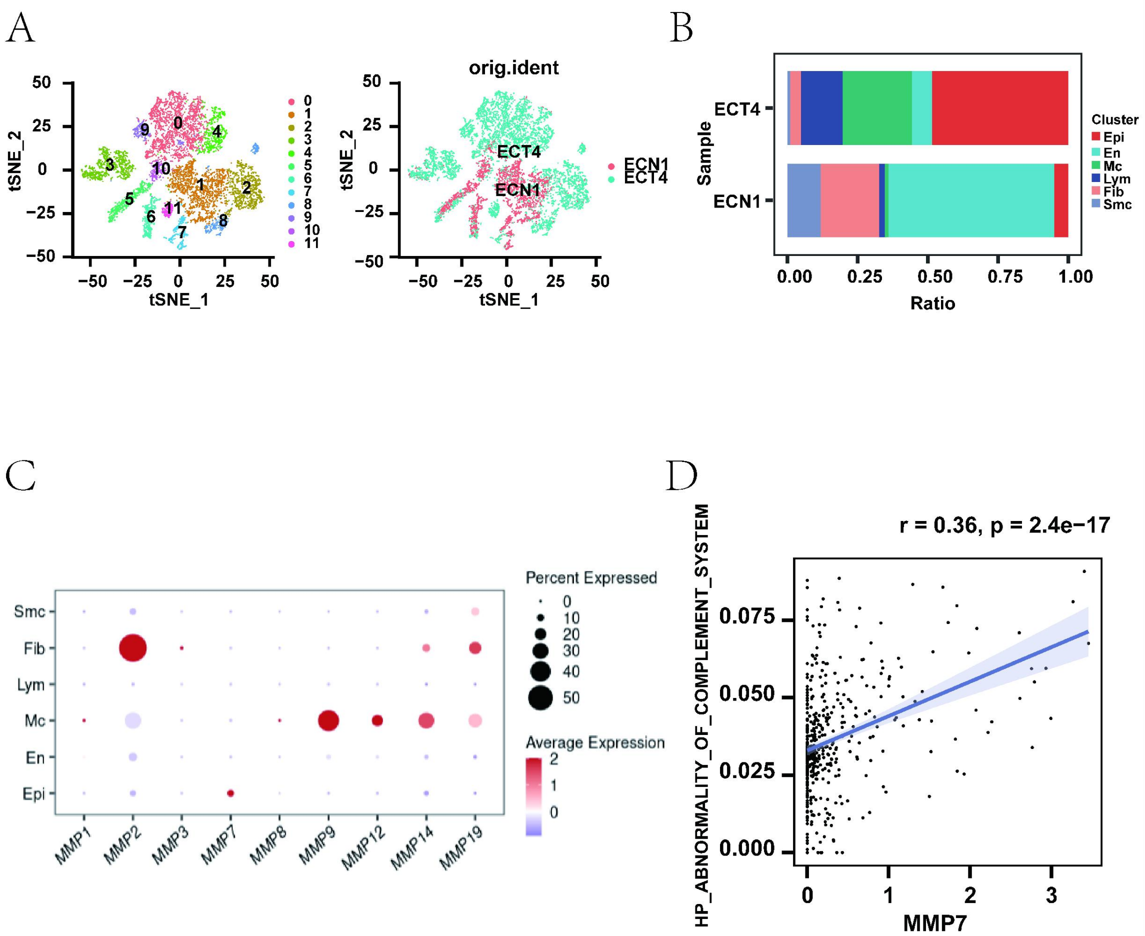
A
orig.ident
tSNE_1
tSNE_2
ECN1
ECT4
B
Sample
Ratio
Cluster
Epi
En
Mc
Lym
Fib
Smc
C
Percent Expressed
Average Expression
Smc
Fib
Lym
Mc
En
Epi
MMP1
MMP2
MMP3
MMP7
MMP8
MMP9
MMP12
MMP14
MMP19
D
r = 0.36, p = 2.4e−17
HP_ABNORMALITY_OF_COMPLEMENT_SYSTEM
MMP7

Supplementary Figure 7. (A) The t-SNE plot demonstrating main cell clusters in normal and EC samples. (B) The cell ratio in normal and EC samples for each cell type. (C) Average expression level of MMP related genes in each cell type. (D) Correlation between the *MMP-7* and abnormality of complement system ($r = 0.36$, $P < 0.001$, Spearman correlation analysis).

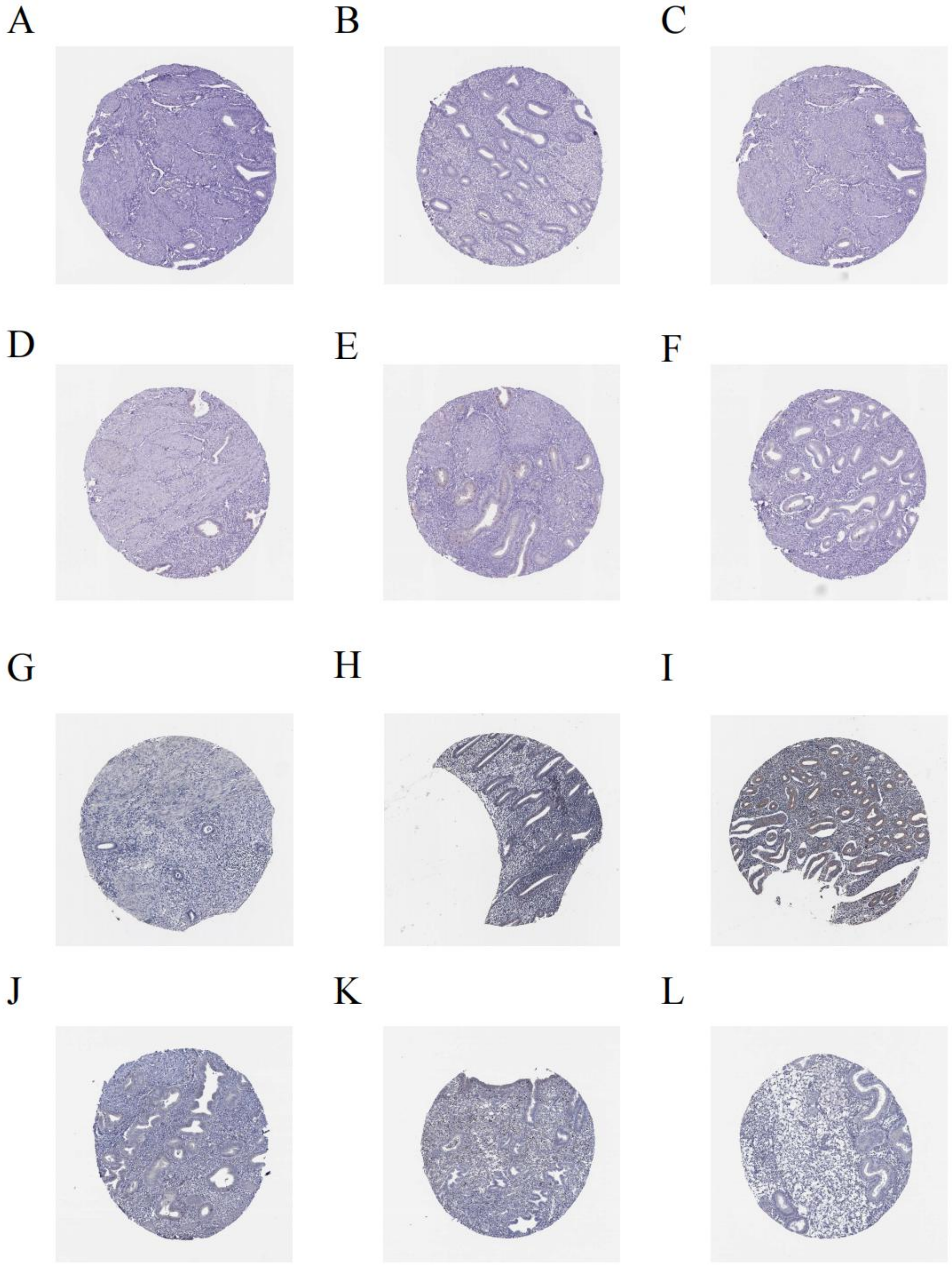
A
B
C
D
E
F
G
H
I
J
K
L

Supplementary Figure 8. Immunohistochemical analysis of MMP expression in EC. (A-C) The expression of *MMP-9* in EC; (D-F) The expression of *MMP-11* in EC. (G-I) The expression of *MMP-15* in EC; (J-L) The expression of *MMP-24* in EC.

Supplementary Table 1. Comparison of basic data from eligible selected endometrial cancer patients and controls.

Patient		Ca			N		
		Ca1	Ca2	Ca3	N1	N2	N3
Characteristic	age	58	56	67	46	54	61
	Time of pregnancy	4	3	1	0	5	3
	parity	3	1	1	0	3	2
	tumor size	2.5*2*1.3	1.5*1.0*0.5	3*2* 1.6	-	-	-
	FIGO staging	II	IA	IIIC1	-	-	-
	histological	Adenocarcinoma	Adenocarcinoma	Adenocarcinoma	-	-	-
	histological grading	High	High	Middle	-	-	-
	lymph node metastasis	Negative	Negative	Positive	-	-	-
	treatment	surgery+radiotherapy	surgery	surgery+chemotherapy	-	-	-

Ca: cancer; N: normal; II: Invasion of cervical stroma with extrauterine extension OR with substantial LVSI OR aggressive histological types with myometrial invasion; IA: Disease limited to the endometrium OR non-aggressive histological type, i.e., low-grade endometroid, with invasion of less than half of myometrium with no or focal lymphovascular space involvement (LVSI) OR good prognosis disease; IIIC1: Metastasis to the pelvic lymph node.

Supplementary Table 1. Comparison of basic data from eligible selected endometrial cancer patients and controls. Including age, times of pregnancy, parity, tumor size, FIGO staging, histological, histological grading, lymph node metastasis and treatment. Ca: cancer; N: normal.
